# Microbial drivers of methane emissions from unrestored industrial salt ponds

**DOI:** 10.1038/s41396-021-01067-w

**Published:** 2021-07-28

**Authors:** Jinglie Zhou, Susanna M. Theroux, Clifton P. Bueno de Mesquita, Wyatt H. Hartman, Ye Tian, Susannah G. Tringe

**Affiliations:** 1 Department of Energy Joint Genome Institute, Lawrence Berkeley National Laboratory, Berkeley, CA USA; 2 Southern California Coastal Water Research Project, Costa Mesa, CA USA; 3 Mount Holyoke College, South Hadley, MA USA; 4 Environmental Genomics and Systems Biology Division, Lawrence Berkeley National Laboratory, Berkeley, CA USA

**Keywords:** Biogeochemistry, Microbial ecology, Soil microbiology

## Abstract

Wetlands are important carbon (C) sinks, yet many have been destroyed and converted to other uses over the past few centuries, including industrial salt making. A renewed focus on wetland ecosystem services (e.g., flood control, and habitat) has resulted in numerous restoration efforts whose effect on microbial communities is largely unexplored. We investigated the impact of restoration on microbial community composition, metabolic functional potential, and methane flux by analyzing sediment cores from two unrestored former industrial salt ponds, a restored former industrial salt pond, and a reference wetland. We observed elevated methane emissions from unrestored salt ponds compared to the restored and reference wetlands, which was positively correlated with salinity and sulfate across all samples. 16S rRNA gene amplicon and shotgun metagenomic data revealed that the restored salt pond harbored communities more phylogenetically and functionally similar to the reference wetland than to unrestored ponds. Archaeal methanogenesis genes were positively correlated with methane flux, as were genes encoding enzymes for bacterial methylphosphonate degradation, suggesting methane is generated both from bacterial methylphosphonate degradation and archaeal methanogenesis in these sites. These observations demonstrate that restoration effectively converted industrial salt pond microbial communities back to compositions more similar to reference wetlands and lowered salinities, sulfate concentrations, and methane emissions.

## Introduction

Wetlands are land areas saturated or covered with water and are a transition zone between dry land (upland) and bodies of water. They are recognized as invaluable ecosystems that perform numerous essential ecosystem services such as improving water quality, regulating climate, mitigating storm surges, and supporting biodiversity [1]. Anoxic conditions in wetland soils slow decomposition and improve the accumulation of organic matter, leading to the potential for carbon (C) sequestration. Vegetated coastal wetlands (e.g., mangrove forests and salt marshes) are particularly effective at taking up carbon dioxide (CO_2_) into plant biomass and organic C in the sediment; this “blue carbon” is proposed as a critical buffer against climate change [2, 3]. The C storage rate of tidal wetlands greatly surpasses that of upland terrestrial ecosystems [4].

While they can be effective C sinks, wetlands are also the single largest natural source of atmospheric methane (~127−155 Tg annually) [5], a greenhouse gas with 25−35 times the global warming potential of CO_2_ over a century timescale [6]. Methane production in wetlands is largely attributed to the activity of anaerobic archaeal methanogens [7]. Anaerobic archaea can produce methane via three pathways—by splitting acetate (acetoclastic methanogenesis), by reducing CO_2_ using hydrogen as an electron donor (hydrogenotrophic methanogenesis), or by demethylating methyl-containing substrates such as methanol, methylamine, or dimethylsulfide (methylotrophic methanogenesis) [8]. The relative importance of these different pathways depends on temperature, organic matter quality, the rates of intermediate processes such as acetogenesis or acetate oxidation, and the presence of alternate electron acceptors such as nitrate, sulfate, or iron, whose reduction competes with methanogenesis to consume acetate and hydrogen [8]. Freshwater wetlands typically have low nitrate and sulfate concentrations, leading to high rates of methanogenesis [9].

Salinization is a widespread and expanding threat to both inland and wetland ecosystems [9]. It is caused by a variety of factors including reduced freshwater inflows, wastewater effluent disposal, sea level rise, storm surges, and road salts [9]. While there is not a specific estimate on the extent of wetland salinization, Wicke et al. 2011 [10] estimated that 1.5 × 10^8^ ha of forests, wetlands, and protected areas were salt-affected. Salinization is expected to cause major changes in wetland C, nitrogen (N), phosphorus (P), sulfur (S), and iron (Fe) cycling. Salinization and the associated increase in sulfate availability have been shown to suppress methanogenesis, as sulfate-reducing bacteria outcompete methanogens for hydrogen leading to the inhibition of methanogenesis [11]. However, the effects may not always be so straightforward, as elevated methane emissions are still observed in saline to hypersaline environments across a wide range of sulfate and dissolved oxygen concentrations [12, 13]. Even in the presence of high sulfate concentrations, methane has been shown to be produced aerobically through methylphosphonate degradation, such that competition with sulfate-reducers is bypassed [12, 14]. Methylotrophic methanogenesis with substrates such as methanol, trimethylamine, and methionine can also proceed uninhibited by high sulfate concentrations [13, 15]. Furthermore, salinization can reduce methane consumption [16], which would serve to increase the net methane emissions.

Industrial salt ponds, created through a process of seawater evaporation, represent the extreme case of wetland salinization, with almost saturated salinities in addition to high light intensity, UV radiation, and elevated temperatures [17]. These extreme conditions greatly limit planktonic biodiversity but support a thriving community of halophilic microorganisms, especially members of the haloarchaea and Chloroflexi [18, 19]. Salt evaporation ponds for salt making are found across the globe, including the San Francisco Bay where large stretches of tidal wetlands were converted to industrial salt ponds over the past century and a half [20, 21]. However, the South Bay Salt Pond Restoration Project is now restoring over 6,000 hectares of former industrial salt ponds to a rich mosaic of tidal and inter-tidal wetlands [22]. The large-scale, multi-phased plan aims to provide wildlife-oriented public access as well as flood management and other ecosystems services in the South Bay. The success of restoration and impacts on biogeochemical cycling and microbial communities has not yet been assessed.

In this study, we sampled sediments from four adjacent wetland sites in the South Bay, including two unrestored former industrial salt ponds, one recently restored pond, and one unaltered remnant (“reference”) salt marsh. We explored the microbial communities in these four wetland sites through both 16S rRNA gene profiling and shotgun metagenome sequencing, in parallel with greenhouse gas measurements and sediment biogeochemical characterization, to investigate the biogeochemical and microbiological effects of restoration. We hypothesized that the restoration activities undertaken to restore the natural hydrology would result in abiotic conditions and microbial communities more similar to reference conditions. Based on previous experiments, we hypothesized that reducing salinity and sulfate levels in the unrestored salt ponds through restoration could lead to increased methanogenesis rates and methane emissions.

## Methods

### Field site and sampling

Former industrial salt ponds R1 and R2 (unrestored, 57.2 and 180.4 ha, respectively), SF2 (restored, 96.9 ha), and reference tidal wetland (20.5 ha, hereafter “R2A”) are located in the Ravenswood complex, Menlo Park, CA, adjacent to the Dumbarton Bridge (Fig. S1). These sites were chosen because they represent three different states of wetland management in the San Francisco South Bay (reference, unrestored, restored) and have been extensively studied as part of previous restoration efforts [23–25]. Additionally, their proximity to each other helps control for broader environmental conditions such as climate. The reference wetland R2A is a tidal salt marsh dominated by cordgrass (*Sporobolus follosus*) and pickleweed (*Sarcocornia pacifica*). The salt-making process started with pumping bay water into the pond system followed by water evaporation inside each pond, leading to a slow concentration of brines for three years [26]; after approximately ~150 years of industrial salt production, salt making discontinued at these sites in 2003 and the ponds were left idle awaiting restoration. SF2 was restored to a managed pond at Bay salinity (22−24 ppt) in 2008 to provide improved flood control, recreation, and wildlife habitat. Restoration work included improving the flood control levee and constructing berms (dividing the pond into two cells), canals (an inlet and outlet to provide uniform water flow through the cells), 30 bird nesting islands, water control structures (to regulate water levels and flow rates of the ponds) and public access features [27].

Soil core samples were collected from each sampling site in July 2014 using a Split Core Sampler fitted with an auger tip (AMS Inc., American Falls, ID). Duplicate soil cores were collected at each of three coring locations (A, B, C) per sampling site, for a total of six cores per site. Duplicate cores were collected adjacent to one another, one core for gas flux measurements and one for DNA and geochemical analyses, and all coring locations were 20−30 m distance from each other, approximately 2 m from the shore. Coring locations at each site were selected to be representative of the site habitat conditions, but a more spatially comprehensive sampling was not possible due to limited site accessibility.

On-site CH_4_ and CO_2_ fluxes were measured from the intact soil cores using a Los Gatos Research Greenhouse Gas Analyzer (GGA; Los Gatos Research, Mountain View, CA). The GGA measures CO_2_ and CH_4_ concentrations at 1 Hz with tunable laser cavity ringdown spectroscopy, at a precision of < 2 ppb (1σ @ 1 Hz) over an operating range of 0.1−100 ppm. Cores were closed on the bottom with airtight caps and placed into 2 L glass Mason jars fitted with airtight Bev-A-Line IV connective tubing (US Plastic Corp, Lima OH) that allowed continuous gas exchange with the GGA’s pumped internal chamber. Soil core fluxes were measured over two consecutive >300 s intervals and ventilated between these cycles for 100 s by gently agitating air above the opened jar while the GGA’s pump purged the gas lines and chamber with ambient air. Time series data on CO_2_ and CH_4_ from the (linear slope) second measurement interval were used to calculate rates of concentration increases within the experimental chamber. Fluxes (µmol m^−2^ d^−1^) were calculated given the volume of the chamber and tubing, and the surface area of the soil core through which gases passed.

Soil core samples were split into two section depths D1 and D2 (0−5 and 5–15 cm belowground, respectively). Cores were homogenized, placed on ice, and frozen at −80 °C for downstream DNA extraction and geochemical analyses. On-site field measurements of water temperature, pH, and dissolved oxygen (DO) were performed with YSI probes (model 6920-v2, YSI Inc., Yellow Springs, OH, USA). Sediments were assessed for total elemental concentrations of C, N, and P; DTPA extractable concentrations of Zn, Mn, Cu, and Fe; 2 M KCl extractable concentrations of nitrate and ammonium; ammonium acetate extractable concentrations of base cations Ca^+^, Na^+^, K^+^, and Mg^2+^; Olsen extractable concentrations of inorganic phosphate; saturated paste extractable concentrations of sulfate and chloride (used as a proxy for salinity [28]); and loss on ignition percentage organic C at the UC Davis Analytical Laboratory. Differences among site types were assessed with Kruskal−Wallis tests followed by Nemenyi posthoc tests.

Duplicate DNA extractions were performed on 1 g homogenized soil from each sample using the MoBio PowerLyzer kit according to the manufacturer’s instructions and stored in the freezer (−20 to −80 °C); both duplicates were individually used for 16S rRNA gene sequencing. For metagenome sequencing, multiple DNA extractions were performed for each sample and pooled to obtain sufficient DNA for the shotgun library construction.

### 16S rRNA gene amplicon processing

16S rRNA gene V4 515F-Y GTGYCAGCMGCCGCGGTAA and 926R CCGYCAATTYMTTTRAGTTT primer sets, modified to include Illumina barcodes and sequencing adapters, were used to PCR amplify the V4 region and the resulting amplicons were pooled and sequenced on a MiSeq (Illumina) [29]. Sequences were then processed through the Joint Genome Institute (JGI) centralized rolling quality control system and iTagger computational pipeline [29] for sequence trimming, clustering operational taxonomic units (OTUs) based on 97% sequence identity, and taxonomic assignment with SILVA version 119 [30], resulting in 3,795 unique OTUs. Functional guilds were assigned from the taxonomy with a semi-automated classification script based on recent literature (Appendix 1). Singletons and potential chimeras were removed using QIIME [29, 31–33]. Mean sequencing depth per sample was 188,358 (± 6560 standard error) reads. OTU counts were normalized with the *DESeq2* R package [34], with normalization based on median of ratios using the functions “estimateSizeFactors”, “estimateDispersions” and “getVarianceStabilizedData” (variance stabilization transformation). Normalized OTU counts were further scaled to a total sum of 1 million in order to obtain relative abundance as counts per million (CPM). 16S rRNA gene sequencing data are available on NCBI; BioProject, BioSample, and SRA accession numbers can be found in Table S1.

### Metagenome sequencing, assembly, annotation, and binning

#### Shotgun metagenome sequencing and data processing

For each metagenome sample, ~20 Gb of shotgun sequence data were generated on the HiSeq 2500 platform (Illumina) in a 2 × 150 paired-end run mode, for a total of 700 Gb with ~400 bp inserts with reads overlapped and merged. The shotgun metagenomic reads were pre-processed using BBTools (filtering and trimming) and assembled using MEGAHIT v1.0.3. The assembled contigs were further processed for annotation and integration into IMG through JGI’s Microbial Genome Annotation Pipeline. The estimated gene copies of metagenomes from all sites were generated by IMG/M (“Compare Genomes” -> “Abundance Profiles Tools”) based on KEGG Orthology Terms (KO ID). The assembled metagenomes are publicly available on IMG and NCBI SRA (accession numbers are listed in Table S1).

#### Genome binning

Each metagenome was re-assembled (*de novo*) using Tadpole error correction in BBTools and the SPAdes assembler to generate longer contigs for high-quality bins [35]. Assembly statistics were obtained from QUAST [36]. Metagenomic reads from each sample were mapped to all contigs using BBTools (https://sourceforge.net/projects/bbmap/) default parameters to get read depths across samples and binning was performed on each individual assembly with three different tools (CONCOCT, MaxBin, and MetaBat), which use different algorithms to cluster contigs into bins based on sequence composition and obtained depth of reads [37–39]. DAS Tool was used to select the best non-redundant set of genome bins from the three software tools [40], and then CheckM was applied to evaluate the completeness, contamination, and strain heterogeneity [41]. Finally, we applied dRep to remove redundant genome bins with high average nucleotide identity (gANI > 96.5% and minimum overlap 60%) across different metagenomes [42]. Only bins with completeness > 70%, contamination < 25% and strain heterogeneity < 200% were retained for downstream analysis, leading to identification of 310 metagenome-assembled genomes (MAGs) used for downstream analyses. Based on the recommendation of Bowers et al. [43], we found 130 MAGs were assigned to ‘High-quality draft’ (> 90% complete and < 5% contamination), 145 were assigned to ‘Medium-quality draft’ (≥ 50% complete and < 10% contamination) and the remaining 35 had completeness from 77 to 100% and contamination from 12 to 24% (Table S2). Raw reads were then mapped to all the collected MAGs using BBTools. The average read depth was used as a proxy for the abundance of genomes and normalized using *DESeq2*. The Spearman correlation of each MAG with methane was also calculated using the *SciPy* Python library (https://www.scipy.org/).

The phylogenetic tree was constructed using genomestoreferencetree [44] based on 56 single-copy genes (SCG) including identified MAGs and reference genomes available in IMG. Taxonomies of newly identified MAGs were assigned based on the nearest two, three, or more neighbors in the phylogenetic tree. Lastly, the Bin Annotation Tool (BAT) was used to assign taxonomies to MAGs based on gene calling, mapping of predicted ORFs against the nr protein database, and voting-based classification of ORFs [45].

### Statistical analyses

#### α-diversity and β-diversity

α-diversity (OTU richness and Shannon index) of 16S rRNA gene amplicon data was calculated based on counts of OTUs rarefied to the minimum library size (140,388 reads per sample) and site differences were assessed with ANOVA. β-diversity was calculated using weighted UniFrac or Bray-Curtis dissimilarity, and site differences were assessed with PERMANOVA using the “adonis” function in the *vegan* R package [46] with 10^4^ permutations and visualized with principal coordinates analysis (PCoA) with the *ggbiplot* R package [47].

#### Indicator species

The *indicspecies* R package was employed to determine indicator OTUs based on the point-biserial correlation coefficient (*r*) of an OTU’s positive association to each individual site type or combination of site types [48]. The resulting OTUs were further analyzed with 10^4^ permutations and considered significant at *p* < 0.001 and *r* > 0.5. Significant OTUs were grouped at the family level and the ten most abundant families for each site type were visualized as a bipartite network representing associations between indicator OTUs and site types using *NetworkX* Python package (https://networkx.org/). Significant OTUs from *indicspecies* were then also validated as differing between any two of the three sites at a false discovery rate corrected *p* value (*P*_FDR_) < 0.001 using likelihood ratio tests implemented in *DESeq2*. Only OTUs significant from both *indicspecies* and *DESeq2* were highlighted using different colors in the co-occurrence networks of all OTUs.

#### Gene copies

The downloaded matrix of the estimated gene copies from annotated metagenomes in IMG was normalized using *DESeq2*, followed by the calculation of Spearman rank correlation for each gene with methane and salinity. The log2 transformations of *DESeq2-*normalized gene counts, as well as their correlations with methane and salinity, were visualized as a heatmap for 149 genes involved in C, N, P, and S cycling [49, 50].

To compare the abundance of phnJ and mcrA genes across the three habitat types and make the difference more biologically meaningful, the estimated gene copies were then normalized using MUSiCC [51]. Final normalized abundances of genes were represented in terms of the fraction of genomes, which included a step of division by the median abundance of 76 universal single-copy genes (USiCGs) used in MUSiCC [51].

#### Co-occurrence networks

Two OTU co-occurrence networks were constructed using the *DESeq2* normalized counts and Spearman rank correlations between OTUs and visualized with the Fruchterman−Reingold layout using *NetworkX*. For the first network (Fig. S2), only strong correlations (Spearman’s |*r*| > 0.9) were visualized. OTUs specific to one or two types of sites were labeled in different colors in the network. The second co-occurrence network includes only OTUs specific to unrestored salt ponds but uses the same correlation matrix as Fig. S2 to highlight interactions within unrestored salt ponds. The top 5% of salt pond-specific OTUs with the highest degree centralities were defined as keystone OTUs of unrestored salt ponds. Keystone OTUs are defined as those with the capacity to drive community composition and function despite relatively low abundance [52].

An additional network (Fig. S3) constructed using 149 unique carbon (specifically methane), nitrogen, phosphorus, and sulfur (CNPS) cycling genes was then used to analyze functional interactions in different sites. Significant and positive correlations between these genes (Spearman’s *r* > 0.7, *P*_FDR_ < 0.05) were selected for visualization. The Girvan−Newman algorithm was applied to define well-connected subnetworks and the optimal assignment was selected based on the highest modularity calculated [53], leading to a total of 19 subnetworks. The five most optimal subnetworks with more than four genes were then visualized separately (Fig. S4).

#### Phylogenetic profiling

The genomes of 173 archaea and 3318 bacteria from IMG were employed for phylogenetic profiling analysis. The presence/absence matrix of the 149 unique CNPS genes in genomes across the phylogenetic tree was downloaded using the function “function profiling” in IMG, followed by calculating correlations between gene pairs. Only the cassettes of genes with Spearman’s *r* > 0.8 and *P*_FDR_ < 0.05 were considered to have significant co-occurrence within a majority of microbial genomes and selected for further analysis.

### Substrate addition experiment

Water collected from Pond R2 (unrestored) was used as a culture medium for a substrate addition incubation experiment. The experiment was conducted in a Type B Vinyl Anaerobic Chamber (Coy Lab Products, Inc. Michigan) enriched with non-flammable hydrogen gas mixture (less than 5%), CO_2_ (5%), and N_2_ (balance). The hydrogen concentration is typically reduced down to 0.2−0.6% during oxygen scavenging and is not enough to support hydrogenotrophic methanogenesis. Consistent with this expectation, minimal methanogenesis was measured in the controls without added substrate. After the water equilibrated overnight in the chamber to remove remaining oxygen, 10−15 mL of soil from an additional core collected from Pond R2 in March 2015 were mixed with 15 mL culture medium to make a soil slurry, which was funneled into autoclaved 100 mL glass bottles. Glass bottles were capped and crimped and stored in the dark inside the anaerobic chamber. Fifteen milliliters solutions of trimethylamine (15 mM), methanol (20 mM), acetate (20 mM), or pure culture medium (control) were prepared and injected into the glass bottles with three replicates for each treatment (*n* = 12 total). CH_4_ production was measured with a Picarro G2508 Cavity Ringdown Spectrometer (Picarro, Inc. California) after substrate addition and every other day for two weeks. Specifically, 10 mL headspace gas was withdrawn from each glass bottle and injected into the Picarro circulation system; 5−10 min after the injection, the circulation system was equilibrated with atmospheric gas so that the recorded gas level was back to atmospheric level before the next sample injection. Each measurement was followed by 10 mL gas addition from the anaerobic chamber to maintain the pressure inside the glass bottle. The amount of methane (dry) in the injection was computed by integrating the area under the curve using the *flux* R package [54]. To estimate the rate of methane production in each soil microcosm, the net methane production was converted to μmol/gram/day by taking into consideration the time elapsed between measurements and the mass of soil inside each bottle. Data were analyzed with repeated-measures ANOVA to test for the effects of time, substrate type, and their interaction.

## Results

### Biogeochemistry and methane flux

**Fig. 1:**
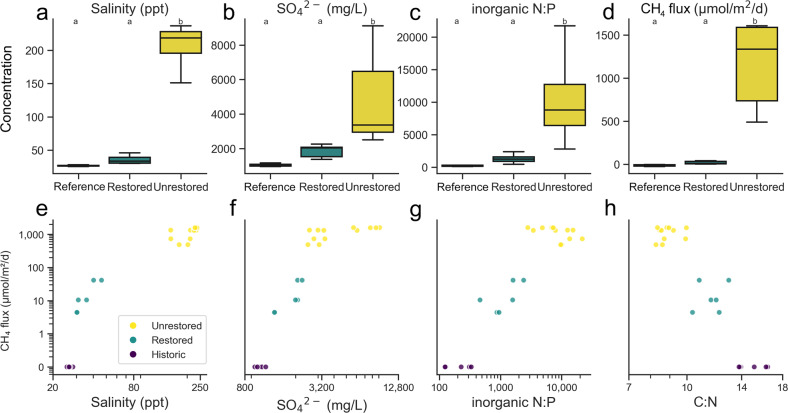
Biogeochemistry of sampled sites. Variation of salinity (Cl^−^) (**a**), sulfate (**b**), inorganic N:P ratio (**c**), and methane fluxes (**d**) in restored/unrestored salt ponds and reference wetland. Methane fluxes were positively correlated with salinity (**e**), sulfate (**f**), and inorganic N:P ratio (**g**) and negatively correlated with C:N ratios (**h**). Note the change in the *y*-axis scale in panels (**a**−**d**) and the logarithmic scale in panels (**e**−**h**). Different letters delineate significant pairwise comparisons (Nemenyi posthoc, *P* < 0.05).

Unrestored salt ponds (“R1” and “R2”) had much higher salinity (range 150.7−236.4 ppt) than the restored salt pond (“SF2”, 30.2−40.2 ppt) and reference wetland (“R2A”, 25.5−28.2 ppt) (Fig. 1a) [55]. The salinities of unrestored salt ponds were roughly five-fold higher than the salinity of common seawater (35 ppt). Unrestored salt ponds had higher sulfate concentrations (2497−9115 mg/L) than the restored salt pond (1367−2249 mg/L) and reference wetland (958−1167 mg/L) (Fig. 1b). Inorganic N:P ratios were also higher in unrestored salt ponds (2798:1−21735:1) than in the restored salt pond (462:1−2394:1) and reference wetland (124:1−332:1) (Fig. 1c) suggesting the availability of P, especially inorganic P, may be limiting in unrestored salt ponds. The abundance of C relative to N was also lower in unrestored salt ponds (Fig. 1h).

Unrestored salt pond cores exhibited significantly higher methane emissions (490.47−1607.09 μmol/m^2^/d) than both the restored salt pond (4.4−41.4 μmol/m^2^/d, Nemenyi test *p* = 0.029) and the reference wetland (−24.3 to −1.7 μmol/m^2^/d, Nemenyi test *p* < 0.001), where negative flux values indicate net methane consumption (Fig. 1d). Methane fluxes were highly correlated (Spearman’s *r* > 0.7) with salinity and sulfate (Fig. 1e, f). Methane was positively correlated with inorganic N:P ratios, but negatively correlated (Spearman’s *r* = −0.83) with C:N ratios (Fig. 1g, h). Additionally, conductivity, chloride, total N:P, calcium, magnesium, sodium, potassium, CO_2_, organic C, organic matter, and zinc were all positively correlated with methane while copper, inorganic phosphorus, dissolved oxygen, and iron were all negatively correlated with methane (*P*_FDR_ < 0.05, Fig. S5).

### Microbial community composition (16S rRNA gene)

**Fig. 2:**
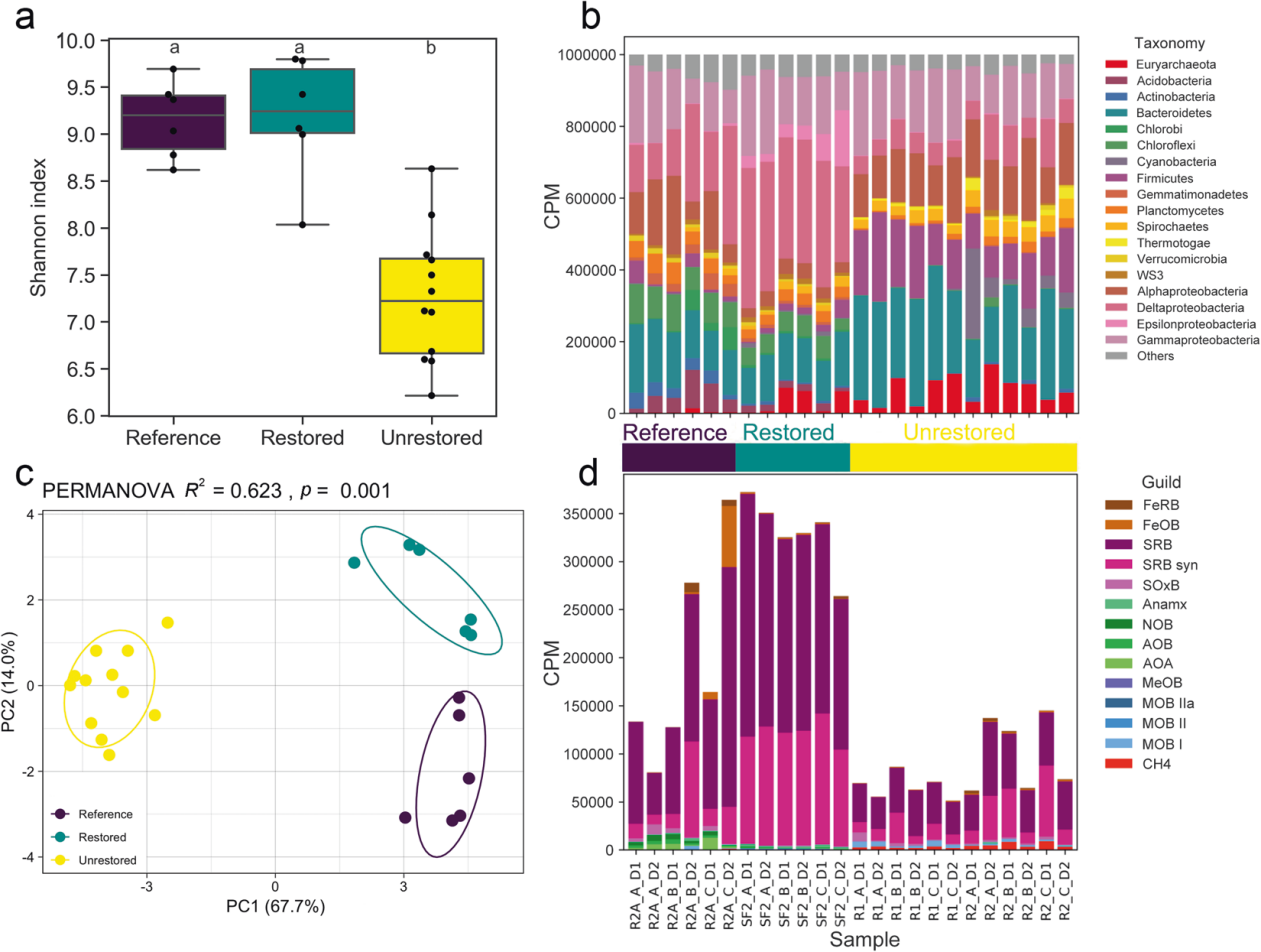
Microbial community diversity and composition from 16S rRNA gene amplicon data. **a** Alpha diversity as measured by Shannon index. **b** Phylum-level community composition (or class for Proteobacteria): counts per million (CPM) following normalization using *DESeq2*’s variance stabilization transformation to account for differences in read depth among samples. **c** PCoA based on the dissimilarity matrix calculated using weighted UniFrac. PERMANOVA confirmed the marked differences among the three types of sites (*R*^2^ = 0.623, *P* < 0.001). **d** Abundance of microbial guilds based on 16S rRNA gene taxonomy and shown as CPM following normalization using *DESeq2’*s variance stabilization transformation to account for differences in read depth among samples. Guilds are: iron-reducing and oxidizing bacteria (FeRB and FeOB respectively); sulfate-reducing bacteria, syntrophs, and sulfur-oxidizing bacteria (SRB, SRB_syn, and SOxB); anammox bacteria (Anamx); nitrite and ammonia-oxidizing bacteria/archaea (NOB, AOB, and AOA); methanol-oxidizing bacteria /non-methanotrophic methylotrophs (MeOB); Type I, II, and IIa methanotrophic bacteria (MOB_I, MOB_II, and MOB_IIa); and methanogens (CH4). Different letters delineate significant pairwise comparisons (Nemenyi posthoc, *P* < 0.05).

Unrestored salt ponds had a significantly lower OTU richness (mean = 1280 ± 49 standard error) and Shannon diversity compared to the restored salt pond (2230 ± 68 OTUs) and reference wetland (1913 ± 115 OTUs) (Fig. 2a, ANOVA, *p* < 0.05). At the phylum/class level, Chloroflexi, Planctomycetes, and Deltaproteobacteria were more dominant in the restored/reference wetlands, while Firmicutes made up a higher percentage of unrestored salt pond communities (Fig. 2b). Microbial community composition was significantly different among the three types of sites, with restored/reference wetland communities more similar to each other than to those of unrestored salt ponds (PERMANOVA *R*^2^ = 0.623, *p* < 0.001), regardless of distance metric used (weighted UniFrac vs. Bray−Curtis; Fig. 2c and Fig. S6). There was no significant effect of depth.

There were 1832 significant indicator OTUs (*p* < 0.001 and *r* > 0.5) out of 3795 total, nearly all of which (1829) were unique to a single site type rather than shared by two site types and absent in the other (Fig. S2a). The co-occurrence network of all OTUs from all three site types (Spearman’s |*r*| > 0.9) indicated that OTUs specific to restored salt ponds and reference wetlands shared more positive correlations compared to those shared with unrestored salt ponds (Fig. S2b).

Indicator taxa in the unrestored salt ponds were largely halophiles, including Rhodobacteraceae, Alteromonadaceae, Balneolaceae, Halanaerobiaceae and Desulfohalobiaceae (Fig. S2a) [56–59]. The restored salt pond included more sulfate-reducers among its indicator species including Desulfobacteraceae, Desulfobulbaceae and Syntrophobacteraceae [60–62]. One OTU (degree = 76, a methanogen, marked with an asterisk) belonging to the order Methanobacteriales was identified as a keystone OTU (top 5% in degree centrality) specific to unrestored salt ponds (Fig. S2c).

Concordant with the indicator species analysis, sulfate-reducing bacteria (SRB) were most abundant in the restored salt pond (Fig. 2d), despite the higher concentrations of sulfate in unrestored ponds (Fig. 1b). Conversely, methanogen abundance was highest in the unrestored salt ponds, as was the abundance of methane-oxidizing bacteria (MOB), though neither of these guilds made up more than 1% of the community in any sample (Fig. 2d). Methanomicrobiaceae, Methanosarcinaceae, and unassigned Methanobacteriales were present in unrestored salt ponds (Fig. S7). Methanosarcinaceae taxa can employ methylamine, methanol, acetate, and H_2_/CO_2_ as methane precursors [63]. Most known Methanomicrobiaceae and Methanobacteriales are hydrogenotrophic; some also use formate, carbon monoxide (CO), or secondary alcohols instead of H_2_ as an electron donor [64]. Ammonia oxidizing archaea and bacteria were highest in reference wetlands, lower in the restored site and nearly absent in unrestored salt ponds; nitrite-oxidizing bacteria (NOB) and anaerobic ammonia-oxidizing (anammox) bacteria displayed a similar pattern, suggesting nitrification and ammonia oxidation may be active in the reference wetland (Fig. 2d).

### Genes positively correlated with methane flux

**Fig. 3:**
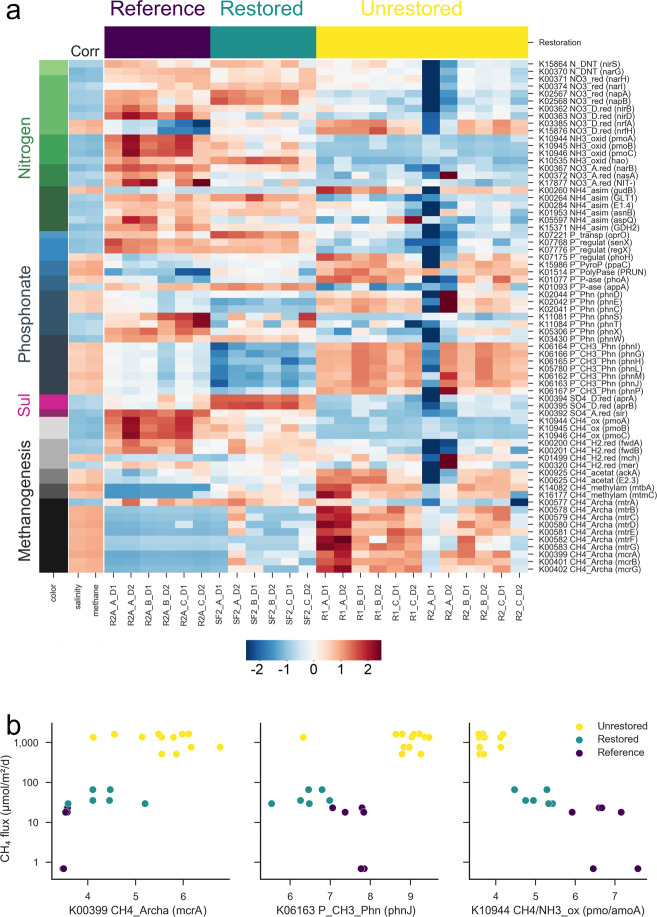
Functional genes for element cycling correlated with methane. (**a**) A heatmap of functional gene abundances in shotgun metagenome data; genes are color-coded by element cycle and pathway, and selected genes are shown, where correlations with methane had Spearman’s *r* > 0.5 from 149 unique CNPS genes. Correlations with CH_4_ fluxes and salinity across all sites are shown in the leftmost heatmap. The central heatmap shows the relative abundance of these genes across sites (values in the heatmap had a step of z-transformation for a better visualization by Seaborn Python package (https://seaborn.pydata.org/), with site type indicated by the bars at the top. The function names of genes were truncated to save space; full names are in Table S3. Scatterplots in (**b**) show relationships between CH_4_ flux and key genes for methanogenesis (*mcrA*), demethylation of phosphonate (*phnJ*) and ammonia/methane oxidation (*amo*/*pmoA*) (*DESeq2* normalized and log2 transformed). Note the logarithmic *y*-axis scale in panel (**b**).

We focused on 149 CNPS genes for in-depth analysis of abundance patterns (Fig. 3 and Fig. S3). Thirty-one of these functional gene families were found to be positively correlated (Spearman’s *r* > 0.5) with methane fluxes across restored and unrestored salt ponds and reference wetlands (Fig. 3a), of which nine are involved in “core methanogenesis” pathways. These include the three genes, mcrABG, encoding methyl coenzyme M reductase, which catalyzes the terminal step in methanogenesis, and the six genes *mtrBCDEFG* comprising tetrahydromethanopterin S-methyltransferase (along with *mtrA* and *mtrH*) for generation of methyl-CoM, the next to last step in hydrogenotrophic and acetoclastic methanogenesis. Five additional genes involved in methane cycling, *ackA*, *pta*, *mtbA*, *mtmC,* and *mch* were more prevalent in unrestored ponds and correlated with methane (Spearman’s *r* = 0.58, 0.59, 0.77, 0.6, and 0.6; Fig. 3a). *ackA* and *pta* metabolize acetate into acetyl-CoA for methanogenesis, *mtbA* and *mtmC* produce enzymes that convert methylamine, an alternative substrate for methanogenesis, to methyl-CoM, and *mch* encodes methenyltetrahydromethanopterin cyclohydrolase which participates in the formation of methane from CO_2_.

The phosphorus cycling genes *phoH* (phosphate starvation-inducible protein), *phoA* (alkaline phosphatase), and *PRUNE1* (exopolyphosphatase) were observed to be most abundant in the salt pond sediments and positively correlated with methane flux, which might reflect a deficiency in phosphate [65] (Fig. 3a). The other two phosphorus cycling genes positively correlated with methane were *ppaC* (manganese-dependent inorganic pyrophosphatase) and *appA* (4-phytase/acid phosphatase). Only three N cycling genes, *nrfAH* encoding nitrite reductase and *gudB* encoding glutamate dehydrogenase (both transform nitrite into ammonia), were significantly and positively correlated with methane (Fig. 3a).

Six genes in one pathway, *phnILHGMJ*, had positive correlations with methane (Spearman’s *r* from 0.56 to 0.64, Fig. 3). These genes encode enzymes involved in the demethylation of methylphosphonates (MPn), which has been demonstrated to generate methane aerobically as a side product through breaking a C–P (phosphonate) bond via the C−P lyase pathway [12, 14, 66]. All genes in this pathway were significantly more abundant in all unrestored salt pond samples than in restored/reference wetland samples. The *phnCDE* genes encoding a phosphonate transport system were also more abundant in unrestored salt ponds and positively correlated with methane (Spearman’s *r* from 0.51 to 0.55, Fig. 3). Notably, *phnJ*, the marker gene of the C–P lyase pathway, was estimated to be present in 14.9% of community members based on single-copy genes (mean genome fraction), about 34 times more abundant than *mcrA* with a 0.4% mean genome fraction (normalized by MUSiCC) in salt pond data (Fig. S8). As these genes are typically present as a single copy, this indicates organisms with the ability to break down phosphonates are at least an order of magnitude more abundant in the salt pond sediments than methanogens.

To identify possible sources of methylphosphonates, we searched for the methylphosphonate synthase gene (*mpnS*, Metcalf et al. 2012; Born et al. 2017) in both sediment and water metagenomes. *mpnS* was found in sediments of reference wetlands and water from both reference wetlands and restored salt ponds, but not in water or sediment from unrestored salt ponds (Fig. S9a, b). The observation suggests that methylphosphonates may accumulate in the sediments of salt marshes while conversion to industrial salt ponds creates conditions favoring their degradation. Upstream genes in the methylphosphonate synthesis pathway such as *pepM*, *E4.1.1.82,* and *phpC* were also more abundant in restored/reference sediments (Fig. S9a), indicating the potential for active transformation of phosphoenolpyruvate (a product of glycolysis) into methylphosphonate [67]. In addition, higher abundance of genes *phnSTUV* and *phnW* (2-aminoethylphosphonate transport system and transaminase) in restored/reference sediments might provide an alternative substrate, 2-aminoethylphosphonate, for methylphosphonate synthesis (Fig. S9a).

### Genes negatively correlated with methane flux

Thirty genes were negatively correlated with methane fluxes. Of particular note were *amo*/*pmoABC* that encode a family of methane and ammonia monooxygenases (Fig. 3a). As *pmoABC* are the key genes for oxidation of methane in methanotrophs, their high abundance in the reference wetlands could explain the lower and even negative values of methane fluxes for those cores despite the higher abundance of phosphonate pathways relative to the restored site (Fig. 3b). An hmm search using all identified *amo*/*pmoA* genes against hmm models of *amoA* and *pmoA* downloaded from FunGene [68] suggested that the majority of these genes in the reference wetland were likely ammonia rather than methane monooxygenases, consistent with 16S rRNA gene amplicon data indicating ammonia-oxidizing microbes significantly outnumber methanotrophs in the reference wetlands (Fig. 2d). One other gene negatively correlated with methane (Spearman’s *r* = −0.69), hydroxylamine oxidoreductase (*hao*), is also found in ammonia-oxidizing bacteria and functions downstream from ammonia oxidation to produce nitrite. Three genes that showed strong and negative correlations with methane, *fwdAB,* and *mer*, are involved in hydrogen metabolism (Spearman’s *r* = −0.67, −0.75, and −0.54) which might indicate a relatively lower activity of hydrogenotrophic methanogenesis in unrestored salt ponds compared to other sites.

Several additional N cycling genes were negatively correlated with methane fluxes (Fig. 3a), including nitrate reductases (*nasA*, *napAB,* and *narBIHG*, transforming nitrate into nitrite), nitrite reductases (*nirABDH* and *NIT-6*, transforming nitrite into ammonia), and nirS (denitrifying nitrite into nitric oxide) as well as three genes *GDH2*, *GLT1*, *GLU*, transforming ammonia to L-glutamate, and additional ammonia assimilation genes (*aspQ*, *asnB*). Most had similar abundance in restored and reference samples, indicating a high potential for N cycling. Only three sulfur cycling genes (*aprAB*, sir) had correlations with methane and all of them were negative. Interestingly, two of these genes (*aprAB*), which are adenylylsulfate reductases involved in sulfate reduction, were relatively more abundant in restored salt pond samples that had higher concentrations of sulfate as compared to reference wetland samples (Figs. 1b, 3a).

### Gene co-occurrence patterns

A correlation matrix of the 149 CNPS genes (*P*_FDR_ < 0.05 and Spearman’s *r* > 0.7) was further used for co-occurrence network analysis (Figs. S3, S4). Five distinct subnetworks with more than four co-occurring genes were identified within the larger network. The almost complete central methanogenesis pathways of *mcrABG* and *mtrBCDEFG*, together with mtbA and *mtmC* involved in digesting acetate, methanol, and monomethylamine, were contained in subnetwork 1, whose abundance was highest in unrestored salt ponds (Fig. S4). While *mtbA* and *mtmC* tend to occur within the same isolate genomes (Spearman’s *r* > 0.8; Fig. S10), the three co-occurring pathways encompassed by subnetwork 1 displayed distinct phylogenetic profiles, indicating they are not trivially co-occurring within genomes and may in fact represent three microbial guilds which cooperate in methanogenesis (Fig. S4).

The phosphonate transport and degradation pathway genes (*phnCDE* and *phnILHGMJ*) were found in subnetwork 3. But these two sets of genes did not consistently co-occur in the network of correlations within IMG reference genomes (Spearman’s *r* > 0.8 and *P*_FDR_ < 0.05, Fig. S11), indicating the existence of microbes in nature with the phosphonate degradation pathway but lacking phosphonate transport genes (Fig. S4). The *phn* genes and an operon (*pstABCS*) encoding the high-affinity inorganic phosphate (Pi) membrane transport system proteins co-occurred frequently in our metagenomic datasets (Fig. S4) but not in reference isolate genomes (Fig. S10), suggesting the two pathways reside in distinct but co-occurring organisms in salt ponds.

The majority of genes from subnetworks 4 and 5 had much higher abundances in the restored site samples than in unrestored or reference samples (Fig. S4). Subnetwork 4 contained sulfate/sulfite reduction genes such as *dsrAB*, *sat*, *met3,* and *aprAB*, consistent with higher populations of sulfate reducers in restored salt ponds (Figs. S4 and 2d). In both subnetworks 4 and 5, we found many methane cycling genes, such as *mtaABC*, *mtbC*, *mttBC*, *mtmB,* and *mtd*, involved in the digestion of methanol, trimethylamine, dimethylamine, and monomethylamine for methanogenesis (Fig. S4). These methane cycling genes were most abundant in restored samples, in contrast to *mcrABG* and *mtrBCDEFG* which were most abundant in unrestored salt ponds. This is consistent with phylogenetic profiling results indicating these genes have distinct patterns of occurrence (Fig. S4, S10). This could indicate an increased rate of microbes digesting dimethylamine and trimethylamine after restoration despite a lower abundance of downstream methanogenesis genes (*mcrABG*). Finally, subnetwork 2 had a mix of genes with higher abundance in both restored and reference samples (Figs. S4) involved in nitrification (*amo*/*pmoABC*, *hao,* and *nxrAB*), nitrate reduction/denitrification (*napAB*, *narBG,* and *nirBDS*), and ammonia assimilation (*GDH2* and *GLT1*), suggesting higher N cycling activity in both site types.

### MAGs connected to methane cycling

**Fig. 4:**
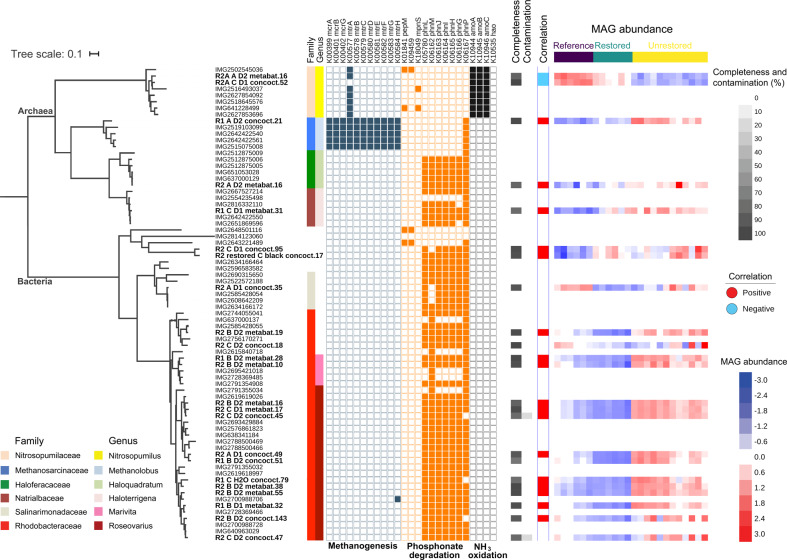
A phylogenetic tree of 23 metagenome-assembled genomes (bolded) with methane generation-related genes and their closest neighbors among IMG reference genomes, constructed based on 56 SCGs. Columns from left to right: family, genus, presence/absence of specific methane cycling genes, percentage of completeness and contamination, correlation with methane (only MAGs with Spearman’s |*r*| > 0.5), and abundance (log2 and then z-score transformed). Note that one MAG (“R2 restored C black concoct.17”) comes from the additional March 2015 unrestored pond R2 sample, while all of the other bolded MAGs are from the main sampling in July 2014.

A phylogenetic tree constructed from MAGs and 2817 representative IMG genomes using 56 SCGs placed our MAGs into at least 24 distinct phyla (Fig. S12). 23 MAGs harboring key genes with potential effects on methane emission, specifically those involved in methanogenesis, phosphonate metabolism, and methane/ammonia oxidation, were selected for further analysis. A new phylogenetic tree was built focusing on these genomes, using the same 56 SCGs and incorporating additional IMG reference genomes of several nearest neighbors belonging to the same genus or family as the MAGs of interest, for more precise classification (Fig. 4). Only one MAG (R1_A_D2_concoct.21) was placed within the methanogenic archaea, grouping with members of the genus *Methanolobus* (family Methanosarcinaceae). This high-quality MAG (Table S2) contained *mcrABG* and its abundance was strongly correlated with methane (Fig. 4; Spearman’s *r* = 0.77 and *P*_FDR_ = 4.11e−06). Most of the genes necessary for methylotrophic, hydrogenotrophic, and acetoclastic methanogenesis were found in this MAG, indicating this organism has the potential to metabolize a broad range of substrates for methanogenesis similar to its nearest neighbors in the *Methanolobus* genus (Fig. 4 and Table S3).

Two *Nitrosopumilus* MAGs with identified *amo*/*pmoABC* genes (completeness 100 and 85.44%, contamination 1.94 and 2.91%, respectively) were strongly and negatively correlated with methane emission (Spearman’s *r* = −0.86 and −0.87, *P*_FDR_ = 8.4e−08 and 4.7e−08), consistent with the amplicon functional guild analysis (Figs. 2d and S6b). This genus is ubiquitous in the oligotrophic ocean surface [69].

20 MAGs harbored the key gene *phnJ* involved in breaking C–P (phosphonate) bonds and generating CH_4_. 15 of these belong to Rhodobacteraceae based on their positions in the tree (11 *Roseovarius*, 2 *Marivita*, 2 not clearly assigned to genus; completeness from 76.06 to 99.70%); this family also contained many indicator OTUs specific to unrestored salt ponds (Figs. 4 and S2c). The majority of *Roseovarius* reference genomes (24 out of 35 in IMG) have the complete phosphonate degradation pathway (*phnILHGMJ*, Fig. 4). While the remaining two MAGs could not be classified to genera due to a lack of close reference genomes, several nearest neighbors including *Roseicyclus mahoneyensis* and two other unclassified Rhodobacteraceae contain the *phnILHGMJ* genes as well (Fig. 4). Another three MAGs with *phnJ* were found to belong to the order Rhizobiales (Fig. 4). One of these fell within the family Salinarimonadaceae, which includes two reference genomes harboring the complete phosphonate degradation pathway, but the closest relatives of the other two Rhizobiales MAGs lacked these genes (Fig. 4). The remaining two MAGs with *phnJ* were halophilic archaea, clustered with reference genomes of *Haloquadratum* and *Haloterrigena*. Many members of these two genera, common in hypersaline environments worldwide, harbored the *phnILHGMJ* genes in their genomes (4 out of 5 *Haloquadratum* and 4 out of 14 *Haloterrigena* reference genomes). Half of all MAGs with *phnJ* were positively correlated with methane flux (Spearman’s *r* > 0.6 and *P*_FDR_ < 0.001).

### Substrate addition experiment

**Fig. 5:**
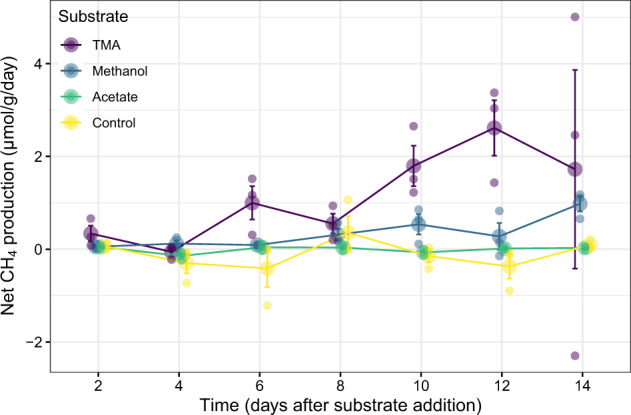
Net daily methane production over two weeks of incubation in an anaerobic chamber with different substrates. Negative values represent net methane consumption. Each substrate had three replicate bottles (small points). Standard error bars are shown around the means (large points). Points were sampled at the same time but are slightly offset (“dodged”) for legibility. TMA = trimethylamine.

Anaerobic microcosms were used to assess the potential for the unrestored salt pond communities to produce methane from different substrates. Substrate type and time significantly affected net methane production rates (repeated measures ANOVA, *p* < 0.05, Fig. 5). Salt pond methanogen populations responded most strongly to additions of trimethylamine (TMA), followed by methanol (but note that TMA has three times the methane production potential as methanol). Acetate addition did not stimulate significant methane production, and control incubations also exhibited little change in methane production rates. The largest increase in CH_4_ production from methanol occurred on day 14, eight days later than the first major increase stimulated by TMA, suggesting changes in the microbial community after long-term incubation with methanol may have contributed. The response to TMA is consistent with the strong and positive correlation of two methylotrophic methanogenesis genes, *mtbA* and *mtmC*, with in situ methane production (Fig. 3a).

## Discussion

Near-coastal saline wetlands are not expected to be substantial sources of methane, as the abundance of sulfate in seawater favors sulfate reduction over methanogenesis. Yet methane emissions from unrestored salt pond sediment cores examined here were comparable to those observed at many freshwater or brackish sites [70], in contrast to our hypothesis. A combination of genetic and taxonomic data coupled with a laboratory incubation experiment suggests that methylotrophic methanogenesis is the primary archaeal methanogenesis pathway in this system. Methanogens were most abundant in unrestored salt ponds, consistent with high methane flux from these ponds and a strong positive correlation between methane flux and core methanogenesis genes (*mcrABG* and *mtrBCDEFG*) across all sites (Figs. 2d and 3a, b). Two methylotrophic methanogenesis genes, *mtbA* and *mtmC*, were observed to have strong and positive correlations with methane flux. The only methanogen MAG assembled from sediment metagenomes was assigned to the genus *Methanolobus* (Methanosarcinaceae) and its abundance was highly correlated with methane flux. Several species from this genus isolated from a variety of environments have been shown to perform methylotrophic methanogenesis [71–73], with some experiments providing evidence for exclusive methylotrophic methanogenesis [74–77]. Furthermore, the substrate addition experiment demonstrated the conversion of trimethylamine and methanol to methane. This is consistent with other studies that have observed methane production in the presence of high sulfate in hypersaline environments, which has been attributed to methylotrophic methanogens’ ability to utilize substrates not accessible to sulfur reducers [12, 14, 78–81]. While these data suggest a primary role for methylotrophic methanogenesis, two lines of evidence also support a potential minor contribution of acetoclastic methanogenesis. First, two acetoclastic methanogenesis genes, *ackA* and *pta*, were strongly and positively correlated with methane. These correlations should be interpreted cautiously, however, because the genes could be present but inactive in the methylotrophic methanogens; indeed, sequenced *Methanolobus* genomes as well as the dominant *Methanolobus* MAG in our data harbor these genes despite the demonstrated preference of this genus for the methylotrophic pathway. Second, sulfate reducers in sediments of unrestored salt ponds were observed to be much less abundant than in restored site samples or reference wetland despite high sulfate concentrations, in contrast to other estuarine and salt marsh samples where methylotrophic methanogenesis and sulfate reduction were found to be simultaneously active [15, 82]. This suggests that despite the high sulfate concentrations, the conditions are not favorable to sulfate reduction and acetoclastic methanogens, therefore, do not face significant competition from sulfate reducers. Our data suggest that hydrogenotrophic methanogenesis is not an important methane production pathway in this system, as three hydrogenotrophic methanogenesis genes, *fwdAB* and *mer*, were negatively correlated with methane (Fig. 3a). In summary, the genetic, taxonomic and substrate incubation data indicate that methylotrophic and possibly acetoclastic methanogenesis are larger contributors to flux in these environments than hydrogenotrophic methanogenesis.

In addition to archaeal methanogenesis, the bacterial phosphonate degradation pathway (*phnILHGMJ*) that demethylates MPn and aerobically generates methane as a side product was observed to have a much higher abundance in unrestored salt ponds than the other two habitats, despite lower dissolved oxygen levels in the unrestored ponds (Fig. 3a, b). An estimated 14.9% of genomes in unrestored salt pond samples harbored phnJ as opposed to 0.4% for *mcrA* (ratio against the median abundance of 76 USiCGs, normalized by MUSiCC), yet the correlation of *mcrA* with methane was stronger (Fig. 3), suggesting these two pathways may jointly contribute to the high methane emission. The C−P lyase phosphonate degradation pathway encoded by *phnILHGMJ* is induced by phosphate starvation in some organisms to take advantage of MPn as a P source [83–86]. This is a common pathway in marine and other phosphate-limited environments to obtain P from organic matter [14, 69, 87–90]. Inorganic N:P ratios in unrestored salt pond sediments were high (Fig. 1c), indicating a deficiency in inorganic P, but total dissolved N:P ratios (11:1−21:1) were close to the average in seawater (16:1), suggesting that taking advantage of organic P would allow resident microorganisms to overcome this limitation, favoring the survival of organisms with the ability to utilize phosphonates. Both community and phylogenetic profiling showed that methanogenesis (*mcrABG* and *mtrABCDEFG*) and phosphonate degradation pathways (*phnILHGMJ*) displayed patterns of occurrence indicating they were harbored by different microorganisms adapted to the conditions in unrestored salt ponds (Figs. S3, S10). Yet another set of genes (*pstABCS*) encoding the high-affinity Pi membrane transport system proteins were significantly correlated with the phosphonate pathway in salt pond metagenomes but not isolate reference genomes, suggesting increased phosphate uptake may be another strategy for surviving the low P availability (Fig. S4). *pstA* has been reported to be significantly and negatively correlated with the concentration of phosphate in ocean surface waters [72], and hence this module is expected to help organisms adapt to P-limitation in unrestored salt ponds by importing more phosphate and scavenging phosphorus from complex molecules.

While up to 21 MAGs contained phosphonate degradation pathways, including both bacteria and archaea, the family Rhodobacteraceae encompasses the majority of these MAGs (15) as well as several OTUs highly enriched in unrestored salt ponds (Fig. S2c). Both observations support the hypothesis that Rhodobacteraceae, especially the genera *Roseovarius* and *Marivita*, are key players in phosphonate metabolism and methane generation in unrestored salt ponds. Despite the limited previous investigation of phylogenetic distribution, the phosphonate pathway is prevalent in the genus *Roseobacter* within the family Rhodobacteraceae (Sosa et al. 2019). However, some Rhodobacteraceae MAGs lacked phosphonate degradation genes and were negatively correlated with methane (Fig. S10), indicating not all Rhodobacteraceae in these communities were able to demethylate methylphosphonates.

The methylphosphonate synthesis gene *mpnS* was not prevalent in sediments of unrestored and restored salt ponds but was observed in the reference wetland (Fig. S9a, b). It was also observed in surface waters from reference and restored sites, particularly the restored sites (Fig. S9b). This implies that methylphosphonate synthesis may be active in both sediments and surface water of undisturbed salt marshes, allowing the accumulation of methylphosphonates in sediments. The nearest neighbor of two *Nitrosopumilus* MAGs from the salt marsh site, *Nitrosopumilus salaria* BD31, contains *mpnS*; therefore, it is possible that the *Nitrosopumilus* in reference wetland could contribute to methylphosphonate synthesis although this gene was not observed in the reconstructed MAGs (Fig. 4). In addition, upstream genes of methylphosphonate synthesis such as *pepM*, phosphonopyruvate decarboxylase (EC4.1.1.82), *phpC*, *phnW,* and *phnSTUV* as well as many MAGs containing these genes were most abundant in the reference wetland (Figs. S9a, S10). The gene *mpnS* is widely distributed in the marine environment [56], consistent with its presence in reference and restored sites that have recently exchanged water with the San Francisco Bay. Isolation and concentration of seawater solutes in salt making apparently lead to depletion of inorganic phosphate and conditions favoring methylphosphonate degradation as opposed to synthesis.

Several N cycling genes, especially those involved in denitrification and ammonia assimilation, were significantly negatively correlated with methane, displaying higher abundance in restored and reference samples (Fig. 3a). This is consistent with previous studies that observed efficient nitrate removal in coastal wetlands while restored salt ponds only gradually regained this ability [91, 92]. Nitrate, like sulfate, is a more energetically favorable electron acceptor than CO_2_; thus, in anoxic sediments denitrification would precede methanogenesis and explain a negative correlation with methane emissions [6, 93]. Lower abundance of genes for denitrification could reflect limited nitrate availability in some samples (Table S4); furthermore, sulfide is known to inhibit nitrate reductases [9], and while we see relatively few sulfate reducers in the unrestored ponds, there may still be sulfide produced. Low concentrations of sulfide may inhibit denitrification and result in low abundance of denitrifiers; and while acetoclastic methanogenesis is also expected to be inhibited by sulfide, as noted above the methanogens present in these sites appear to be capable of using multiple substrates so inhibition of acetoclastic methanogenesis may not lead to reduced abundance of methanogens or methanogenesis genes. In addition, the *amo*/*pmoABC* genes were highly negatively correlated with methane (Fig. 3a, b). Other lines of evidence including hmm search results, co-occurrence with *hao* and other nitrification genes, and 16S rRNA gene data revealing abundant ammonia oxidizers in the reference site (Fig. 2d) indicated these genes were more likely ammonia monooxygenases rather than methane monooxygenases. Even so, some evidence suggests that the functional boundary of *amoABC* and *pmoABC* is not explicit and ammonia-oxidizing bacteria can also oxidize methane, still indicating the potential possibility of methane consumption in the reference wetland. Indeed, greater rates of anaerobic methane oxidation (consumption) by sulfate-reducers [6] is another possible explanation for the lack of methane emissions in the reference wetland. This is consistent with the finding of greater abundances of sulfate reducers and net consumption of methane in the reference wetland. It is possible that the high salinity of the unrestored salt ponds reduced methane oxidation, as has been shown in other studies [16].

The construction of industrial salt ponds involves feeding seawater and drawing out water through natural evaporation, leading to a highly concentrated brine. This can lead to high concentrations of sulfate, yet salt pond sediments from South San Francisco Bay harbored lower overall populations of sulfate reducers and higher populations of methanogens than a reference wetland or a restored salt pond. This trend was driven by unassigned genera in the Desulfuromonadales order and the Desulfobacteraceae, and Desulfobulbaceae families, which we speculate are not halotolerant, and while some halophilic genera were more abundant in the unrestored salt ponds, these genera represented a very small proportion of the community (< 1%). The hypersaline and phosphate-stressed conditions of salt pond sediments selected for salt-loving microbes such as Rhodobacteraceae, many of which possessed genes for phosphorus scavenging via methylphosphonate degradation, which can produce methane as a side product. Restoration of salt ponds back to wetlands greatly lowered salinities, sulfate concentrations, and methane emissions, increased C:N ratios, and restored microbial communities taxonomically, phylogenetically, and functionally; together, these metrics indicate restoration may have climate benefits due to mitigation of GHG emissions, as well as ecological benefits due to habitat restoration.

## Supplementary information

Supplementary Information (Figures S1−S12; Appendix 1)

Table S1: Sample IDs and accession information

Table S2: checkM results showing completeness, contamination, and strain heterogeneity of the 310 MAGs

Table S3: Table of 532 carbon, nitrogen, phosphorus, and sulfur cycling KOs

Table S4: Sample metadata and biogeochemical data
