# Supplementary material for: Microbial drivers of methane emissions from unrestored industrial salt ponds
Source: ISME J. 2021 Jul 28;16(1):284–95. doi: 10.1038/s41396-021-01067-w (PMC8692437; doi:10.1038/s41396-021-01067-w)
Supplement: Supplementary file 1 — Supplementary Information (Figures S1−S12; Appendix 1) [file 41396_2021_1067_MOESM1_ESM.pdf]

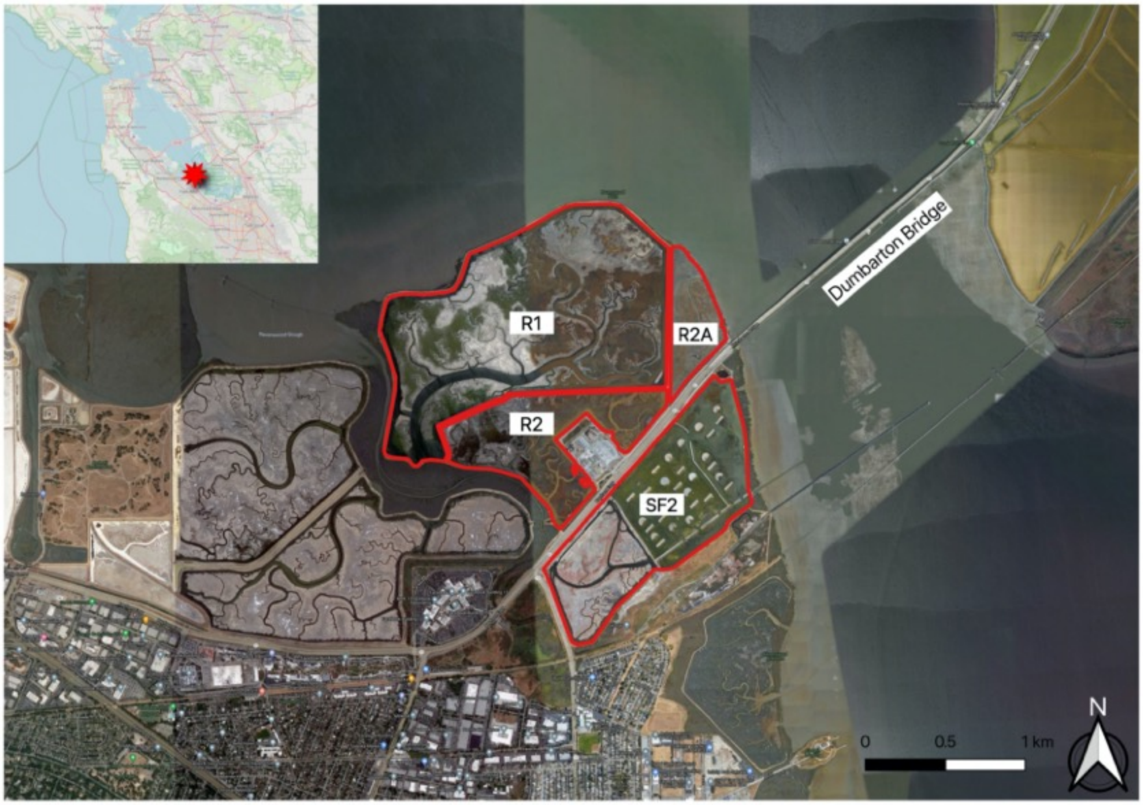

Figure S1. Map of the study sites in the South San Francisco Bay, California, USA (37.49˚N, 122.13˚W). Ponds R1 and R2 are unrestored industrial salt ponds, SF2 is a restored industrial salt pond, and R2A is a reference tidal wetland.

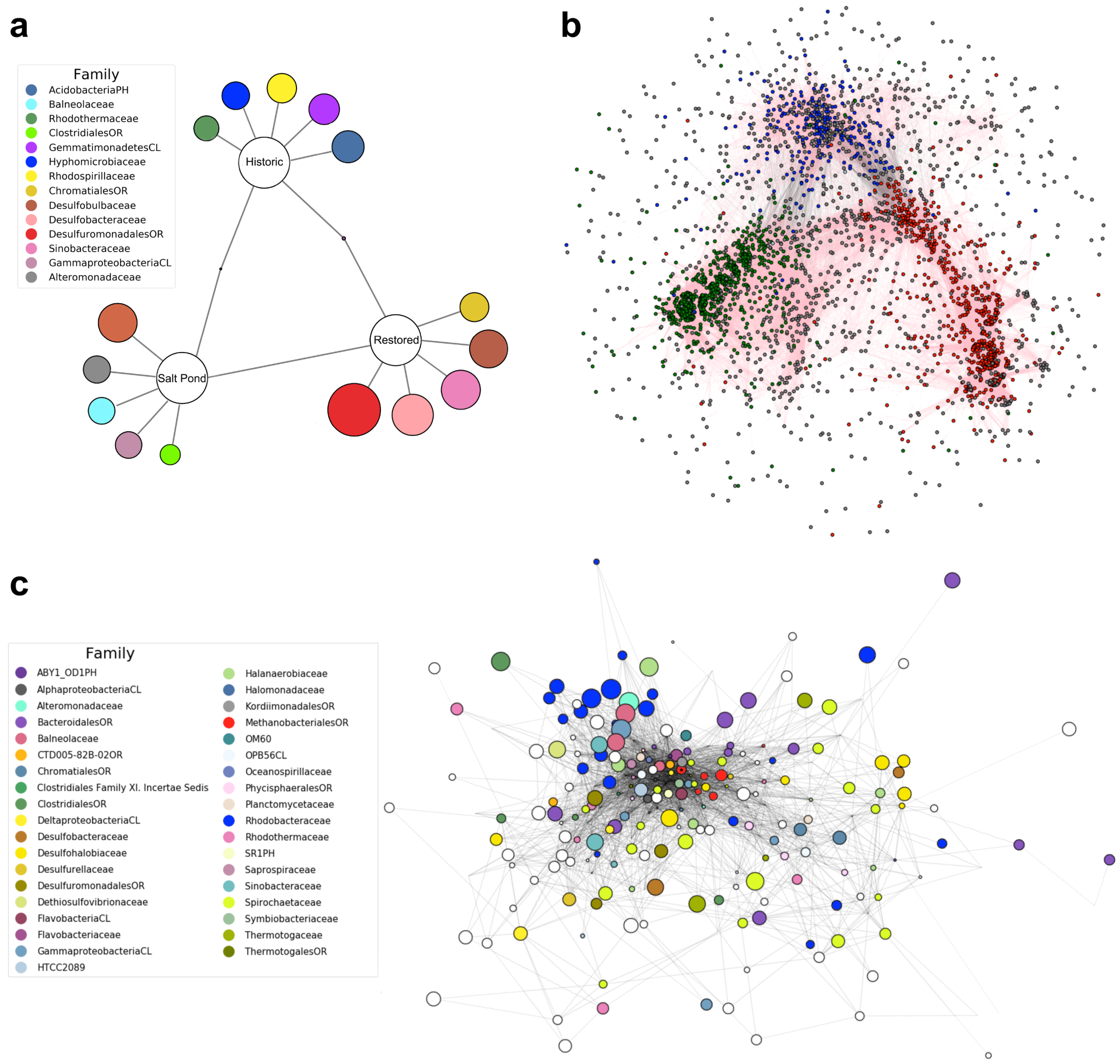

Figure S2. (a) A bipartite network displaying indicator OTUs grouped at the level of family that are positively and significantly associated ($p < 0.001$ and $r > 0.5$) with one or more of the three habitats. The size of each node is proportional to mean OTU abundance (DESeq2 normalized counts) for each type of habitat. Only the 5 most abundant families per site type are shown. (b) The co-occurrence networks of all OTUs were constructed using the DESeq2 normalized counts and conducted Spearman rank correlations between OTUs and visualized with the Fruchterman-Reingold layout using *NetworkX*. Only strong correlations (Spearman's $|r| > 0.9$) were visualized in order to reduce complexity. Only indicator OTUs significant from both *indicspecies* and *DESeq2* were labelled in different colors in order to represent their uniqueness to each or two types of sites. The red indicated historic wetlands, the green indicated restored salt ponds and the blue indicated industrial salt ponds. (c) The co-occurrence network of only OTUs that were defined as site-sensitive OTUs of industrial salt ponds was constructed using the matrix of *DESeq2* normalized counts and conducted Spearman rank correlations between OTUs and visualized with the Fruchterman-Reingold layout using *NetworkX*. The node sizes were corresponding to means of *DEseq2*-normalized counts in unrestored salt ponds.

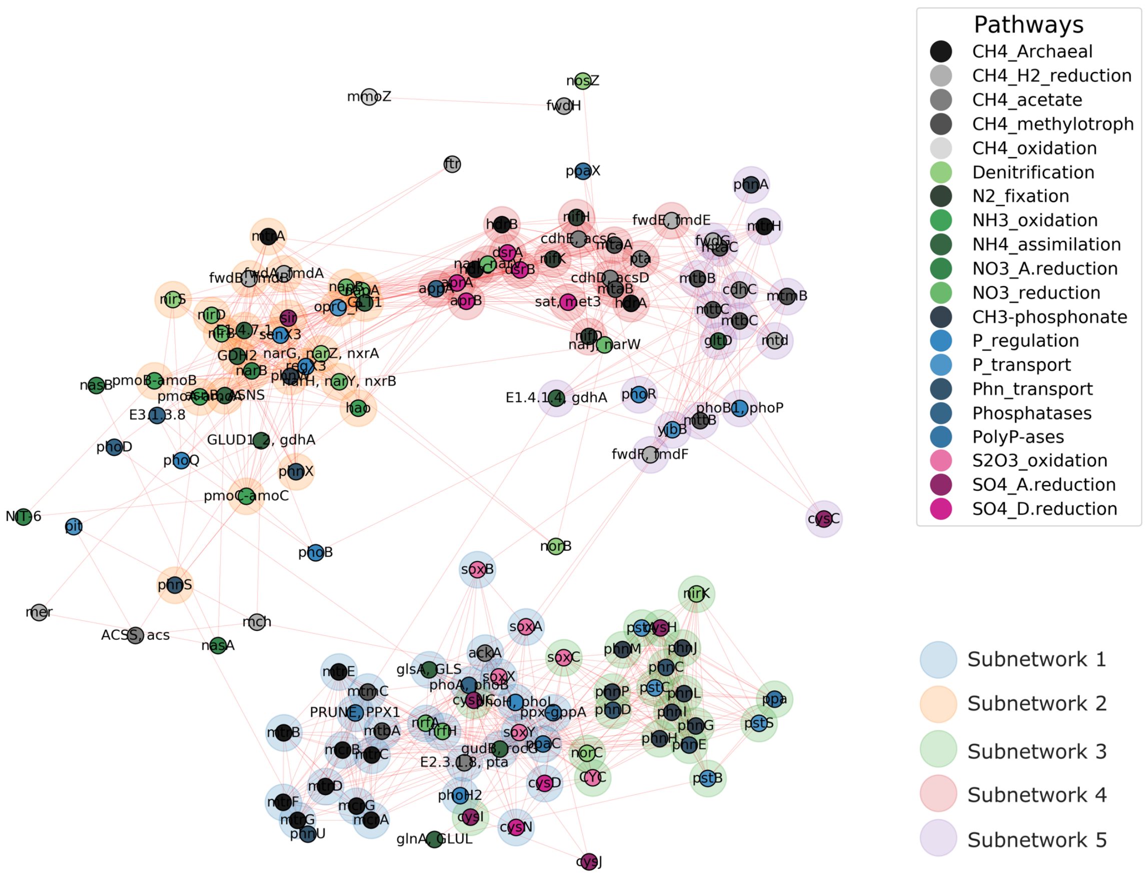

Figure S3. Gene occurrence network constructed using 149 unique CNPS genes and visualized with the Fruchterman-Reingold layout using *NetworkX*. The significant and positive correlations between these genes with Spearman's $r > 0.7$ and $P_{FDR} < 0.05$ were selected for visualization. The nodes representing each gene in the network are labeled with gene names and colored based on their pathway associations, with surrounding halos colored according to subnetwork assignment. The Girvan-Newman algorithm was applied for discovering subnetworks and the optimal assignment was selected based on the highest modularity calculated.

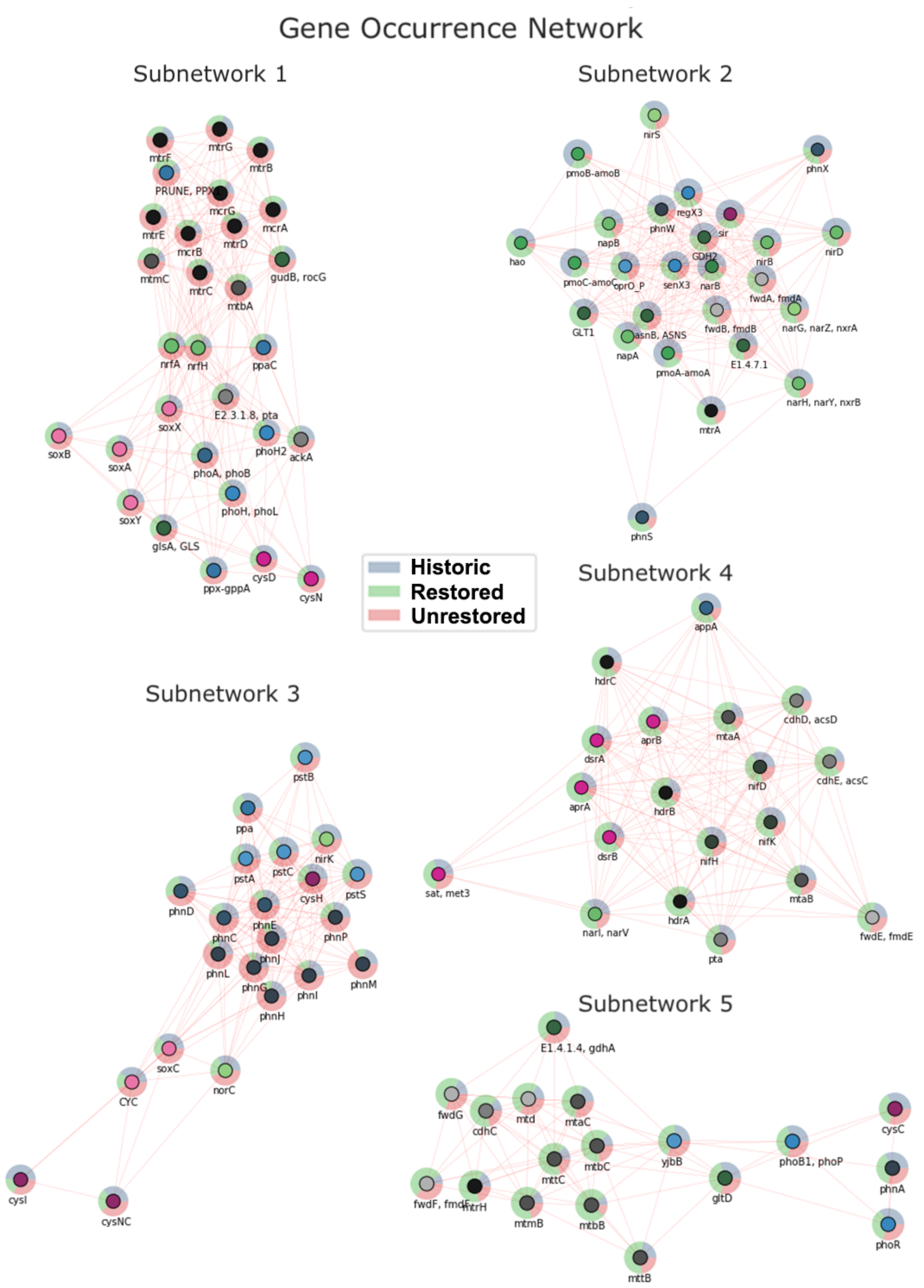

Figure S4. The five optimal subnetworks that were selected using the Girvan-Newman algorithm (shown in Figure S5) and visualized separately with the Fruchterman-Reingold layout using *NetworkX*. The nodes are labeled and colored as in Figure S5. The shadows behind nodes are pie charts indicating relative abundance in historic wetlands and restored and unrestored salt ponds (blue, green and red respectively).

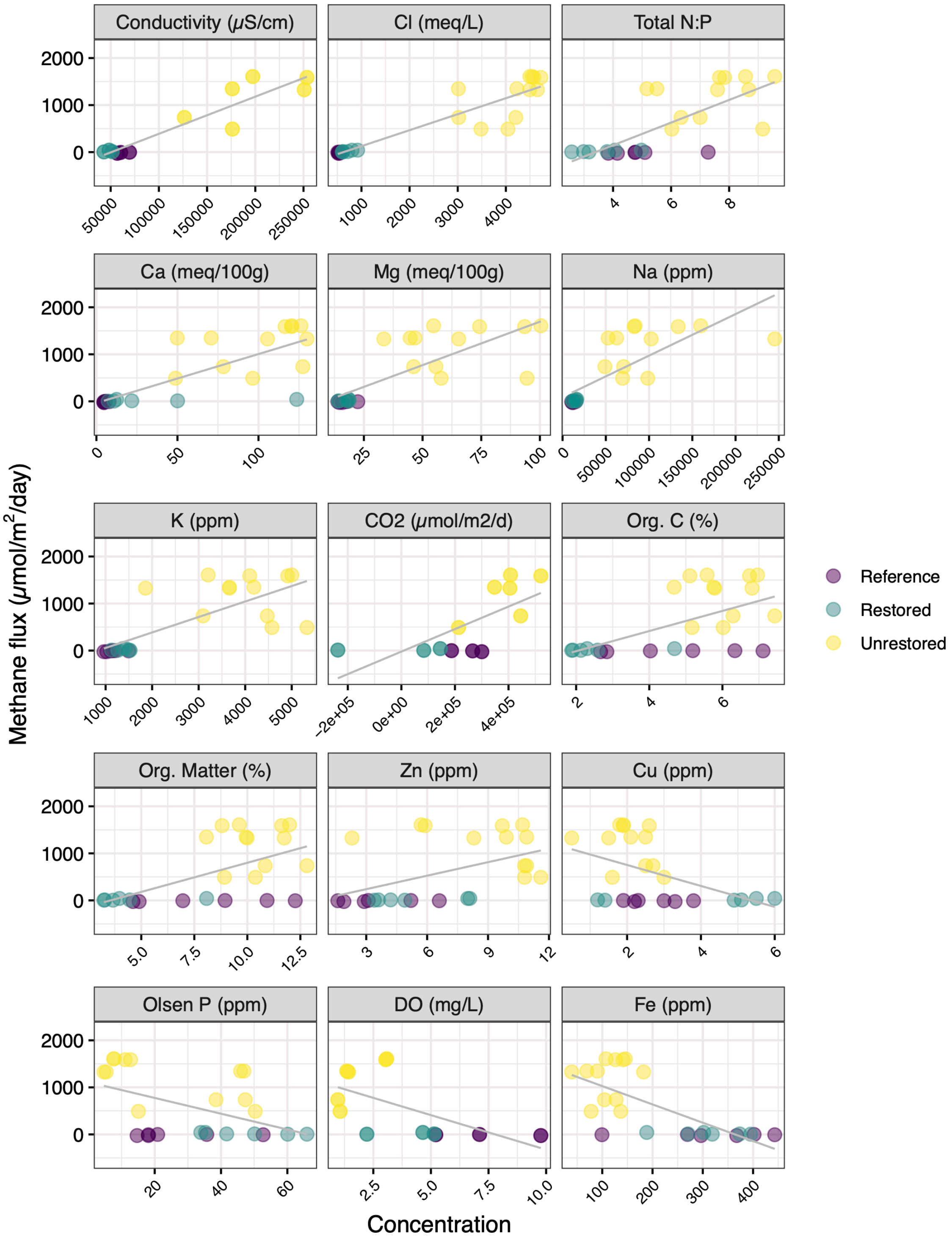

Figure S5. Significant correlations (Spearman, $P_{FDR} < 0.05$) between methane and various chemical variables.

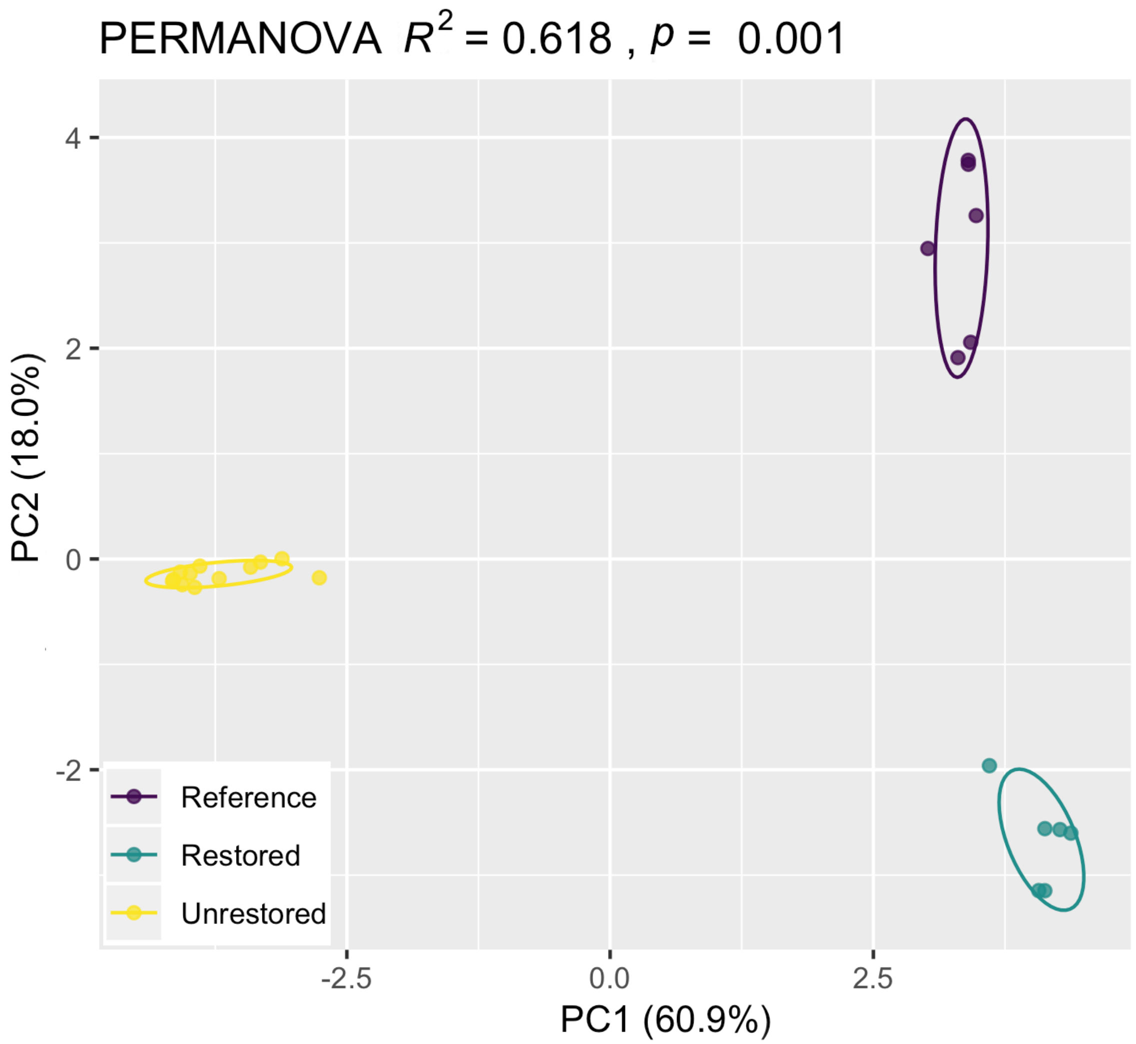

Figure S6. PCoA analysis of 16S rRNA amplicons based on Bray-Curtis dissimilarity. PERMANOVA confirmed the marked difference between the three types of sites (R^2 = 0.618, $P < 0.001$).

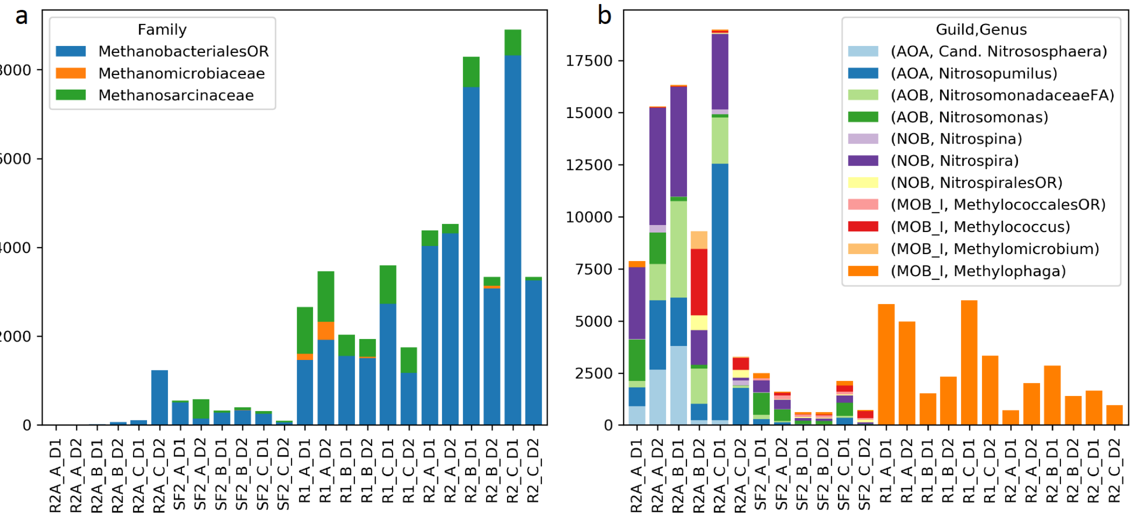

Figure S7. Abundance of microbial guilds of methanogens (a) and ammonia oxidizing bacteria/archaea and methanotrophic bacteria (b) based on 16S rRNA taxonomy and shown as counts *DESeq2* normalized and log2 transformed.

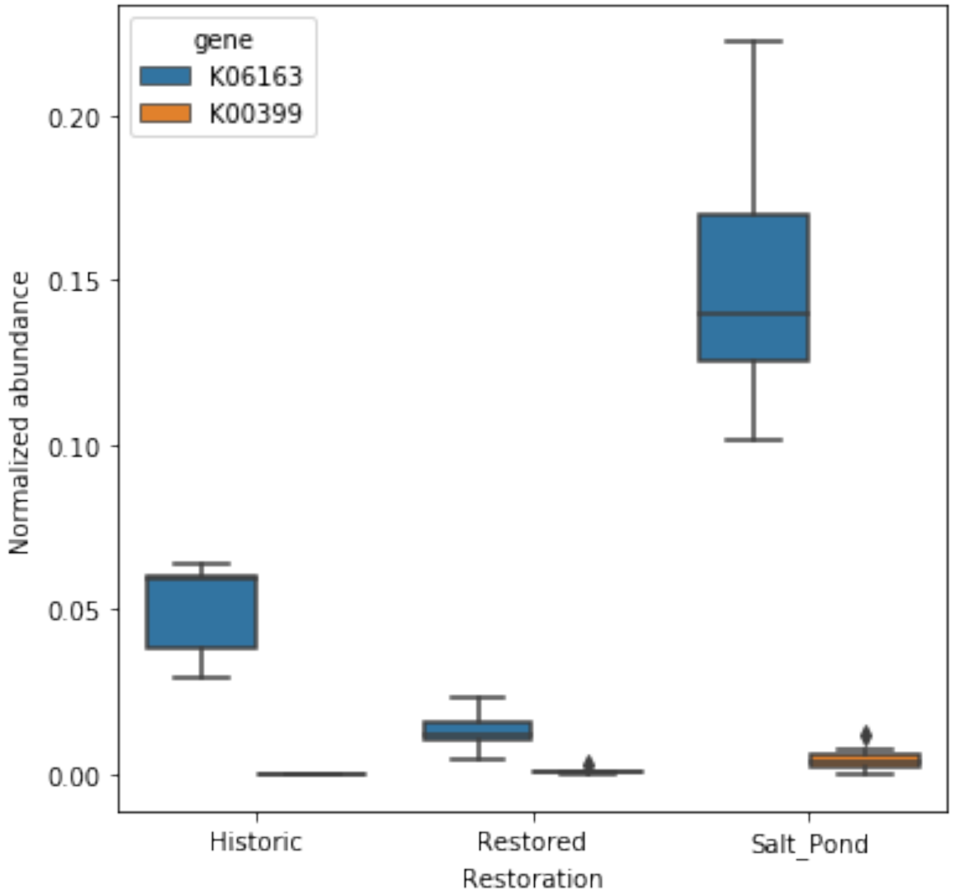

Figure S8. The abundance of phnJ (K06163) and mcrA (K00399) across three types of habitats. The estimated gene copies were normalized using MUSiCC, which corrected these biases of abundance based on universal single-copy genes and machine learning methods.

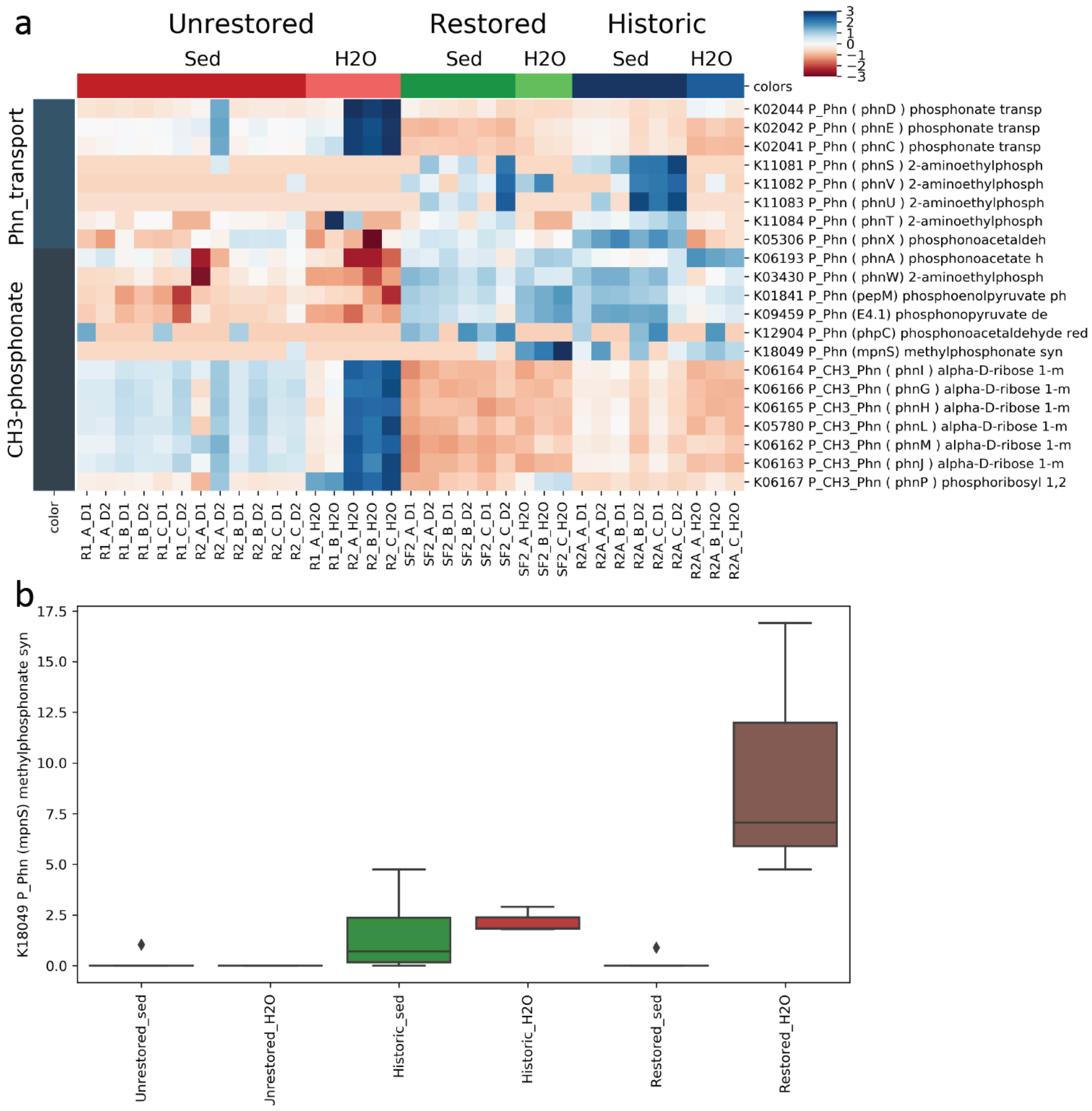

Figure S9. a) Functional genes abundance (*DESeq2* normalized counts with z-score normalization) of phosphonate pathway and methylphosphonate synthesis in both sediments and surface water samples. b) Boxplot of mpnS abundance (*DESeq2* normalized and log2 transformed) averaged across sample type.

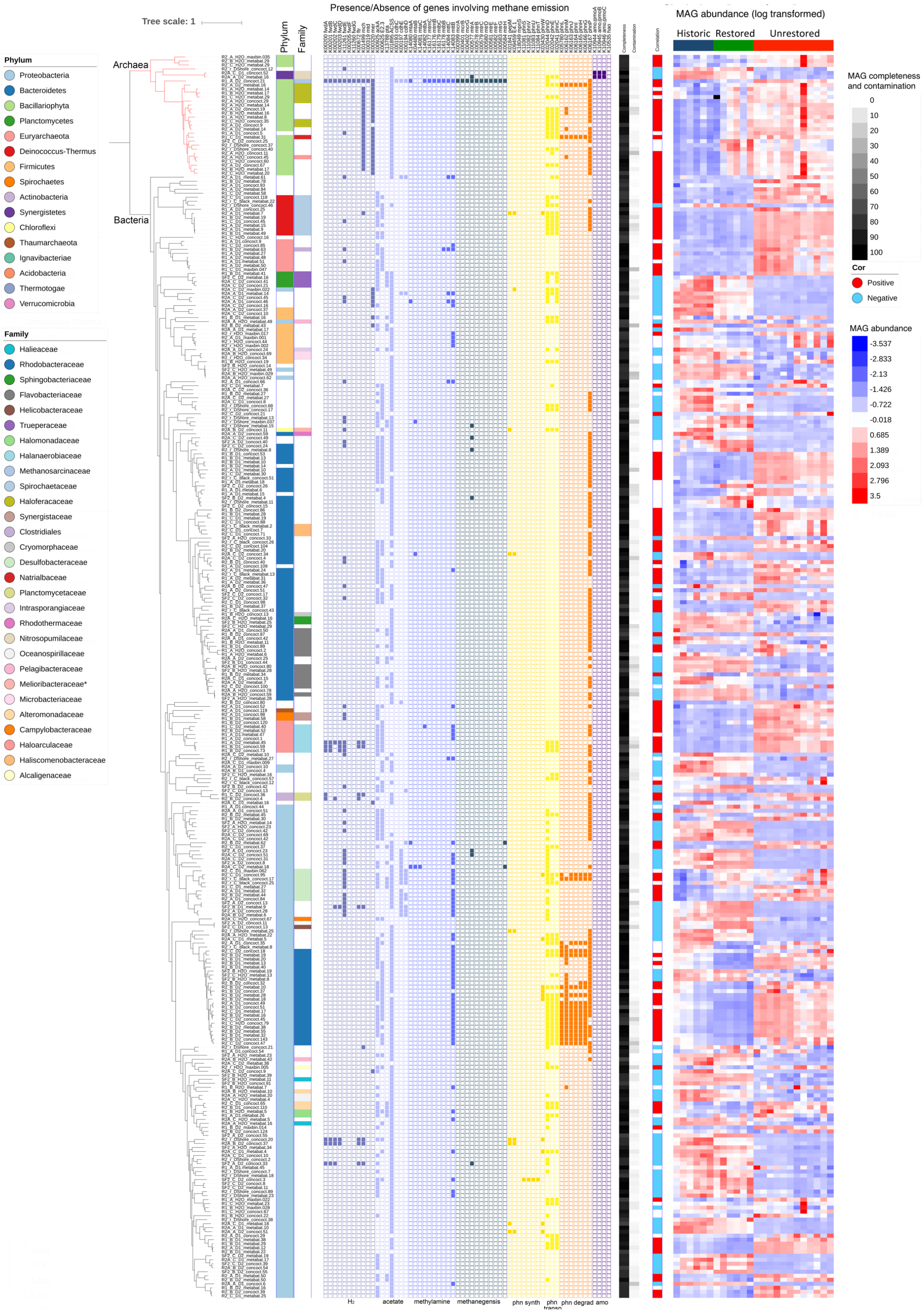

Figure S10. The phylogenetic tree constructed based on 56 SCG single copy genes including only 310 bins with methane generation related genes, associated with the presence/absence of these genes, percentage of completeness/contamination, bins' correlation with methane (only bins with Spearman's $|r| > 0.5$) and MAG abundance (log2 and then z-score transformed). The taxonomy columns (Phylum and Family) were predicted and assigned using the Bin Annotation Tool, BAT (only more than 50% predicted ORFs assigned to the category).

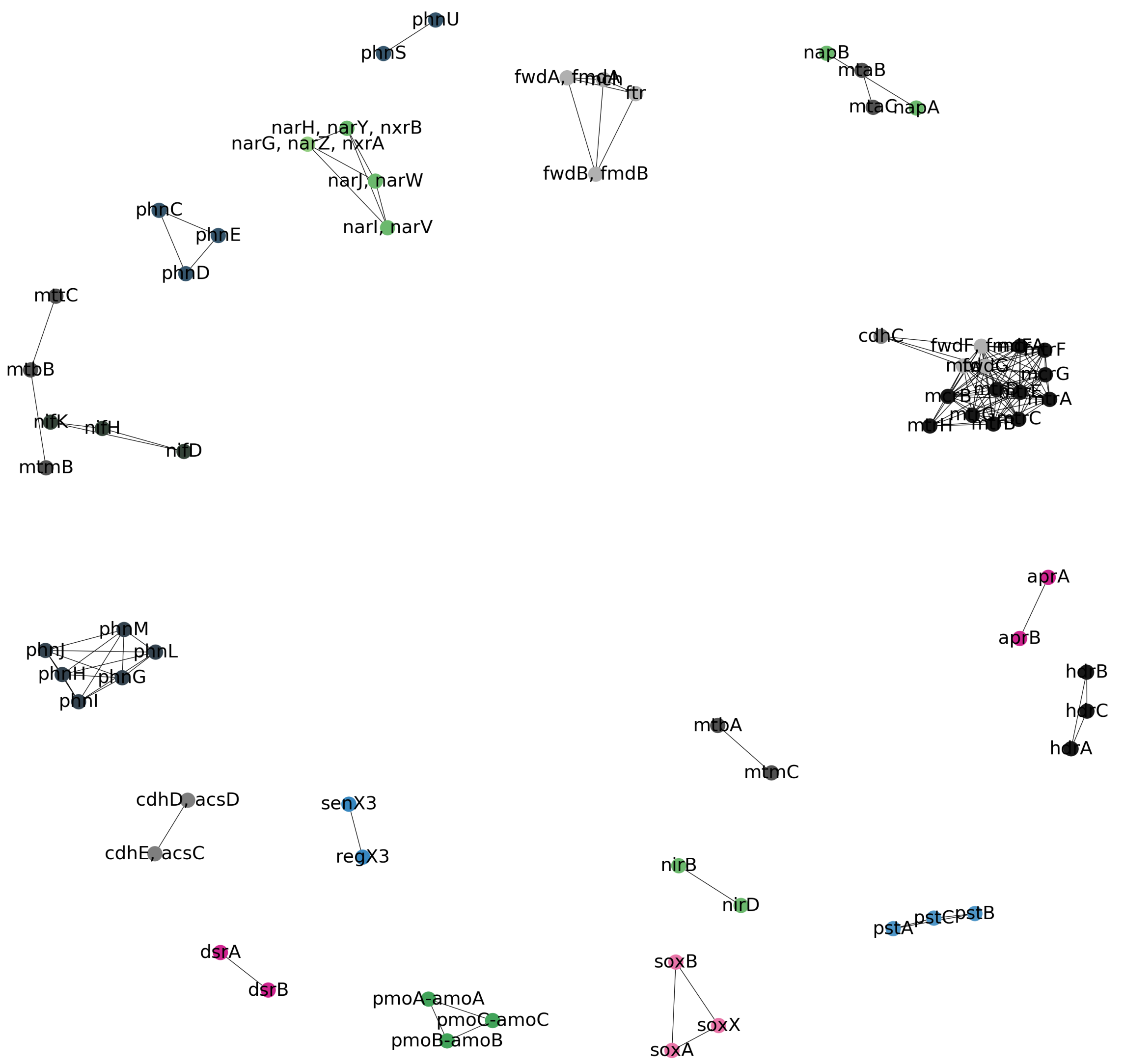

Figure S11. The gene occurrence network including 149 unique CNPS genes of 173 bacteria and 3318 archaea from IMG were subjected to phylogenetic profiling analysis. Correlations were calculated using the presence/absence matrix of the 149 unique CNPS genes in genomes. Only the cassettes of genes with Spearman's $r > 0.8$ and $P_{FDR} < 0.05$ were considered to have significant co-occurrence within the majority of microbial genomes and selected for visualization.

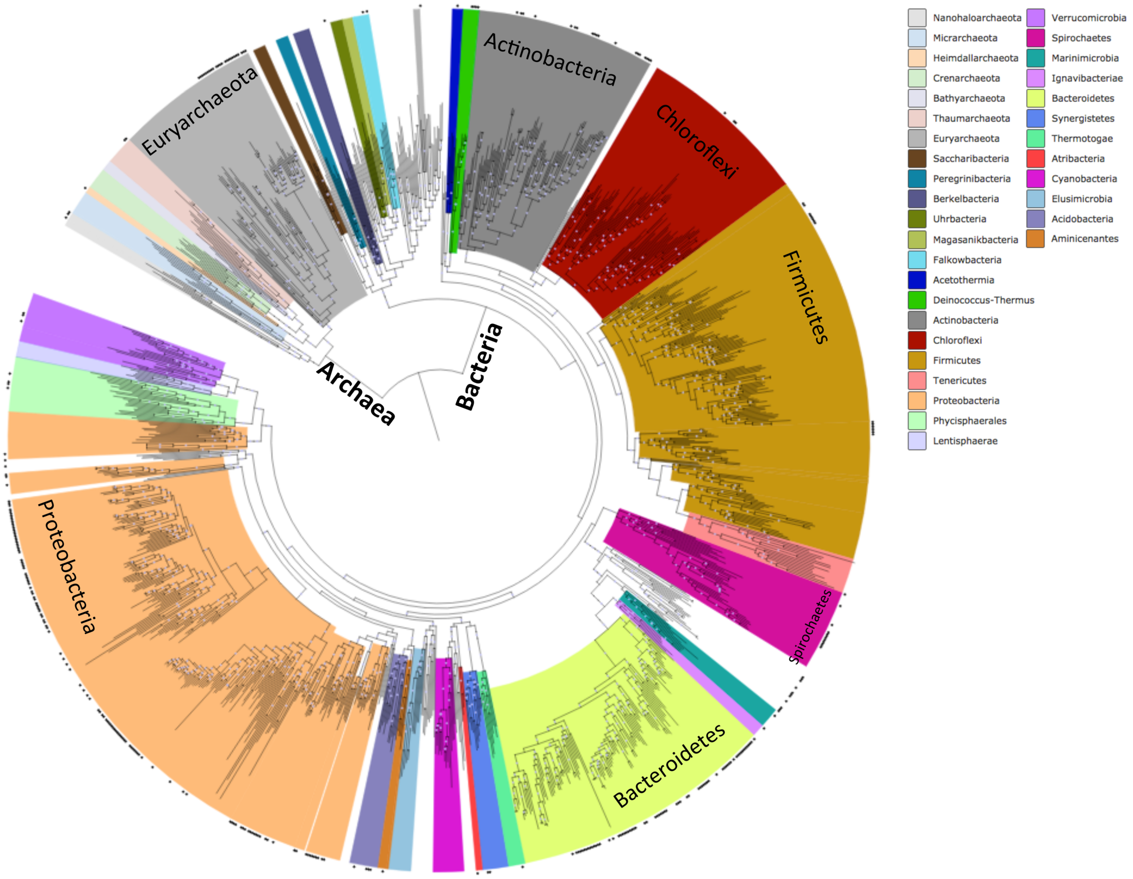

Figure S12. The phylogenetic tree constructed using 56 single copy genes shows that the 310 MAGs are classified into at least 24 phyla. The MAGs from this project are indicated using black dots.

Appendix 1: Functional guild assignments for CH_4, N, S, and Fe cycling from 16S rRNA taxa

Microbial functional guilds were assigned based on 16S rRNA taxonomic identities where biogeochemical functions could be ascertained from taxa names. While the authors recognize the inherent limitations of this approach, these functions were assigned where literature reviews indicated functions are relatively monophyletic, or where taxonomic naming conventions are applied consistently. Guild assignments were applied based on taxonomic strings in OTU abundance tables, filtering only organisms matching search terms using grep searches for particular taxa names. Search terms and supporting literature for each microbial guild are described in detail below, and the code to derive these guild abundances from OTU tables is available on github (functions in section 3 of this code).

Methane production and oxidation

Methanogenesis is a monophyletic function carried out exclusively by archaeal taxonomic groups. Abundances of methanogens were obtained by filtering OTU tables according to assigned taxonomy, using the search term "Methano" across taxonomic ranks. This naming prefix appears present in dominant methanogen classes (*Methanobacteria*, *Methanococci*, *Methanopyri*), orders, families, and genera (Hedderich and Whitman 2013, Nazaries et al. 2013). However, some newly discovered methanogen groups would not be recovered by this search term, like the candidate phylum *Bathyarchaeota* (Bräuer et al. 2020). Within results for methanogenic taxa, a further search was conducted for the order "Methanosarcinales" to approximate the abundances of "acetoclastic" methanogens, while other groups were labeled as "hydrogenotrophic." This classification scheme is an approximation, recognizing that some acetoclastic organisms may also be capable of hydrogenotrophy or methylotrophy (e.g. Methanosarcinacea), while other Methanosarcinales do not use acetate or hydrogen (Hedderich and Whitman, 2013). However, this scheme separates exclusively hydrogenotrophic methanogens from the "acetoclastic" group, while recognizing that methylotrophic methanogenesis does not appear to be monophyletic (Nazaries et al. 2013, Bräuer et al. 2020).

Methanotrophic microbial groups were identified from published reviews, and divided into functional guilds based on monophyletic taxonomic groupings (Hedderich and Whitman 2013, Nazaries et al. 2013). Nearly all genera of methanotrophs have names beginning with the string "Methylo" (Knief 2015, Khadka et al. 2018), which was used to filter (grep) taxonomic abundance tables for methanotrophs. A notable exception to this naming convention are *Verrucomicrobia* organsims, named with the prefix "Methyla," which would not be captured by the initial search term. Guilds of methanotrophic taxa were delineated by further taxonomic subdivision, with search filtering for class-level taxa "Gammaproteobacteria" used to identify Type I methanotrophs, and "Alphaproteobacteria" used to filter Type II methanotrophs, which were further subdivided to separate Type IIa methanotrophs using the search term "Methylocystaceae" on family-level taxonomic assignments (Knief 2015, Khadka et al. 2018). A further class level filter for "Betaproteobacteria" was used to remove methylotrophs from the original "Methylo" search, as this class contains true

methylotrophs (e.g. utilizing methanol) like those in the family *Methylophilaceae* (Chistoserdova 2015, Chistoserdova et al. 2009, Smith and Wrighton 2019). Finally, methane consumption may also be carried out by anaerobic methane-oxidizing archaea, which follow a taxonomic naming convention of “ANME” (Wang et al. 2014) which was used as an additional search filter.

Nitrifiers and anammox

Some microbial nitrogen cycling functions like nitrification and anamox (anaerobic ammonia oxidation) appear to be phylogenetically constrained. Nitrifying bacteria can be classified into functional guilds including ammonia oxidizers (including bacteria and archaea, or AOB and AOA), nitrite oxidizers (NOB), and anammox bacteria (in the *Planctomycetes*) (Bouskill et al., 2012). Significantly, nitrifying bacteria follow a taxonomic convention where nitrite oxidizers are named with the prefix “Nitro” and ammonia oxidizers with the prefix “Nitroso”, except for anammox bacteria. This naming convention is broadly applicable to AOA (Alves et al., 2018), AOB, and NOB (Bouskill et al., 2012), although it may not capture certain Actinobacterial AOB (Khadka et al. 2018). The search term “Nitros” was first used to obtain nitrifying bacteria and archaea, which were then split using an additional search for “Nitroso” to separate ammonia oxidizers. An additional “Archaea” search term at kingdom level taxonomy was applied to separate AOB from AOA. Anammox bacteria were obtained by searching for genus names of the few confirmed genera, including “Kuenenia”, “Anammoxoglobus”, “Scalindua”, “Brocadia”, and “Jettenia” (Cai et al. 2020).

Sulfate reduction, oxidation, and syntrophs

Sulfate reducing organisms were identified following published comparative analyses and reviews (Plugge et al. 2011, Müller et al. 2015), and enumerated using grep – based filtering of taxonomic abundance (OTU) tables. Many sulfate reducers have taxa names beginning with “Desulf,” which was used a primary search (grep) term. However, not all organisms with these names in taxonomic strings are sulfate reducers, and organisms containing the strings “Nitros” (*Nitrospinaceae*), “Syntroph” and “Geobacter” (both treated as separate guilds) were removed from the initial search results. Yet, several other taxa known to reduce sulfate do not indicate this term in their names, including bacteria and archaea. Additional search terms (Müller et al. 2015) included "Caldiserica”, “Thermanaeromonas”, “Sporomusaceae”, “Carboxydothermus”, “Pelotomaculum”, “Moorella“, “Ammonifex”, “Acetonema”, “Thermosinus”, “Thermanaeromonas”, “Carboxydothermus”, “Caldiserica”, “Gordonibacter”, “Thermodesulfobium”, “Thermodesulfovibrio”, and “Magnetobacterium”, along with sulfate reducing archaea “Archeoglobus”, ”Pyrobaculum”, ”Vulcanisaeta”, and ”Caldirvirga”.

Sulfur oxidizing organisms were identified following (Müller et al. 2015), with many organisms containing a taxonomic string “Thio.” Other organisms not containing this name were obtained using the search (grep) terms “Allochromatium”, “Marichromatium”, “Halochromatium”, “Alkalilimnicola”, “Halorhodospira”, “Ruthia”, “Vesicomyosocius”, “Sedimenticola”, “Sideroxydans”, “Sulfuricella”, “Riegeria”, “Azospirillum”, “Rhodomicrobium”, “Magnetospirillum”, “Magnetococcus”, “Chlorobium”, “Chlorobaculum”, and “Prosthecochloris".

Syntrophic bacteria are closely related to sulfate reducers, though some may have lost the capability for sulfate reduction (Plugge et al. 2011). The search term "Syntroph" was used to identify these organisms, given many are found in the order *Syntrophobacterales*, with three families (Plugge et al. 2011). However, other groups of syntrophic bacteria may exist which would not be obtained by this search (Sieber et al. 2012, Worm et al. 2014).

Iron reduction and oxidation

Iron reducing bacteria were enumerated by a search filter for common environmental iron reducers, using the terms "Geobacter", "Shewanella", "Thermoanaerobacter", "Deferribacter", "Geothrix", and "Albidiferax". Notably, this is not a comprehensive list of taxa capable of iron reduction (Schröder et al. 2003, Hori et al. 2015). Iron oxidizing bacteria were identified using search terms obtained from review papers (Hedrich et al. 2011, Ilbert and Bonnefoy 2013, Kato et al. 2015), including the common name fragments "Ferro", "Lepto", and "Metallo", along with additional taxa including "Mariprofundus", "Gallionella", "Sideroxydans", "Acidithiobacillus", "Rhodopseudomonas", and "Sulfolobus".

References

Alves, R.J.E., Minh, B.Q., Urich, T., von Haeseler, A., and Schleper, C. (2018). Unifying the global phylogeny and environmental distribution of ammonia-oxidising archaea based on amoA genes. Nat Commun *9*, 1517.

Bouskill, N.J., Tang, J., Riley, W.J., and Brodie, E.L. (2012). Trait-based representation of biological nitrification: model development, testing, and predicted community composition. Front. Microbio. *3*, 364.

Bräuer, S.L., Basiliko, N., M. P. Siljanen, H., and H. Zinder, S. (2020). Methanogenic archaea in peatlands. FEMS Microbiology Letters *367*, fnaa172.

Cai, M., Ye, F., Wu, J., Wu, Q., Wang, Y., and Hong, Y. (2020). Bias of marker genes in PCR of anammox bacteria in natural habitats. PLoS ONE *15*, e0239736.

Chistoserdova, L. (2015). Methylotrophs in natural habitats: current insights through metagenomics. Applied Microbiology and Biotechnology *99*, 5763–5779.

Chistoserdova, L., Kalyuzhnaya, M.G., and Lidstrom, M.E. (2009). The expanding world of methylotrophic metabolism. Annu. Rev. Microbiol. *63*, 477–499.

Hedderich, R., and Whitman, W.B. (2013). Physiology and biochemistry of the methane-producing archaea. In The Prokaryotes, E. Rosenberg, E.F. DeLong, S. Lory, E. Stackebrandt, and F. Thompson, eds. (Berlin, Heidelberg: Springer Berlin Heidelberg), pp. 635–662.

Hedrich, S., Schlömann, M., and Johnson, D.B. (2011). The iron-oxidizing proteobacteria. Microbiology *157*, 1551–1564.

Hori, T., Aoyagi, T., Itoh, H., Narihiro, T., Oikawa, A., Suzuki, K., Ogata, A., Friedrich, M.W., Conrad, R., and Kamagata, Y. (2015). Isolation of microorganisms involved in reduction of crystalline iron(III) oxides in natural environments. Front. Microbiol. *6*.

Ilbert, M., and Bonnefoy, V. (2013). Insight into the evolution of the iron oxidation pathways. Biochimica et Biophysica Acta (BBA) - Bioenergetics *1827*, 161–175.

Kato, S., Ohkuma, M., Powell, D.H., Krepski, S.T., Oshima, K., Hattori, M., Shapiro, N., Woyke, T., and Chan, C.S. (2015). Comparative genomic insights into ecophysiology of neutrophilic, microaerophilic iron oxidizing bacteria. Front. Microbiol. *6*, 1265.

Khadka, R., Clothier, L., Wang, L., Lim, C.K., Klotz, M.G., and Dunfield, P.F. (2018). Evolutionary history of copper membrane monooxygenases. Frontiers in Microbiology 2493.

Knief, C. (2015). Diversity and habitat preferences of cultivated and uncultivated aerobic methanotrophic bacteria evaluated based on pmoA as molecular marker. Frontiers in Microbiology *6*, 1346.

Müller, A.L., Kjeldsen, K.U., Rattei, T., Pester, M., and Loy, A. (2015). Phylogenetic and environmental diversity of DsrAB-type dissimilatory (bi)sulfite reductases. ISME J *9*, 1152–1165.

Nazaries, L., Murrell, J.C., Millard, P., Baggs, L., and Singh, B.K. (2013). Methane, microbes and models: fundamental understanding of the soil methane cycle for future predictions: Methane, microbes and models. Environ Microbiol *15*, 2395–2417.

Plugge, C.M., Zhang, W., Scholten, J.C.M., and Stams, A.J.M. (2011). Metabolic flexibility of sulfate-reducing bacteria. Frontiers in Microbiology *2*, 81.

Schröder, I., Johnson, E., and de Vries, S. (2003). Microbial ferric iron reductases. FEMS Microbiol Rev *27*, 427–447.

Sieber, J.R., McInerney, M.J., and Gunsalus, R.P. (2012). Genomic insights into ayntrophy: the paradigm for anaerobic metabolic cooperation. Annual Review of Microbiology 429–452.

Smith, G.J., and Wrighton, K.C. (2019). Metagenomic approaches unearth methanotroph phylogenetic and metabolic diversity. In Methylotrophs and Methylotroph Communities, (Caister Academic Press), pp. 57–84.

Wang, F.-P., Zhang, Y., Chen, Y., He, Y., Qi, J., Hinrichs, K.-U., Zhang, X.-X., Xiao, X., and Boon, N. (2014). Methanotrophic archaea possessing diverging methane-oxidizing and electron-transporting pathways. ISME J *8*, 1069–1078.

Worm, P., Koehorst, J.J., Visser, M., Sedano-Nunez, V.T., Shaap, P.J., Plugge, C.M., Sousa, D.Z., and Stams, A.J.M. (2014). A genomic view on syntrophic versus non-syntrophic lifestyle in anaerobic fatty acid degrading communities. Biochimica et Biophysica Acta *1837*, 2004–2016.
